# Plant-type pentatricopeptide repeat proteins with a DYW domain drive C-to-U RNA editing in *Escherichia coli*

**DOI:** 10.1038/s42003-019-0328-3

**Published:** 2019-03-01

**Authors:** Bastian Oldenkott, Yingying Yang, Elena Lesch, Volker Knoop, Mareike Schallenberg-Rüdinger

**Affiliations:** IZMB – Institut für Zelluläre und Molekulare Botanik, Abt. Molekulare Evolution, University of Bonn, Kirschallee 1, 53115 Bonn, Germany

## Abstract

RNA editing converting cytidines into uridines is a hallmark of gene expression in land plant chloroplasts and mitochondria. Pentatricopeptide repeat (PPR) proteins have a key role in target recognition, but the functional editosome in the plant organelles has remained elusive. Here we show that individual *Physcomitrella patens* DYW-type PPR proteins alone can perform efficient C-to-U editing in *Escherichia coli* reproducing the moss mitochondrial editing. Single amino acid exchanges in the DYW domain abolish RNA editing, confirming it as the functional cytidine deaminase. The modification of RNA targets and the identification of numerous off-targets in the *E. coli* transcriptome reveal nucleotide identities critical for RNA recognition and cytidine conversion. The straightforward amenability of the new *E. coli* setup will accelerate future studies on RNA target recognition through PPRs, on the C-to-U editing deamination machinery and towards future establishment of transcript editing in other genetic systems.

## Abstract (web summary)

Bastian Oldenkott et al. show that single moss pentatricopeptide repeat proteins with a DYW domain are sufficient to drive efficient C-to-U RNA editing in Escherichia coli. They demonstrate that the *E.coli* system is an easy to manipulate platform for future studies on RNA target recognition and C-to-U RNA editing.

## Introduction

C-to-U RNA editing of organellar messenger RNAs (mRNAs) is nearly omnipresent in land plants and can affect up to thousands of sites in chloroplast or mitochondrial transcriptomes, respectively^1,2^. Core to the recognition of specific cytidines for conversion into uridines are plant-specific RNA-binding pentatricopeptide repeat (PPR) proteins^1,3,4^. PPR arrays in these editing factors are of the PLS type with long (L, 35–36aa) and short (S, 31–32aa) PPR variants alternating with the canonical P-type PPRs of 35 amino acids. The three carboxy-terminal PPRs of PLS-type PPR proteins generally differ in their amino acid conservation and are labeled P2, L2, and S2. Many additional *trans*-acting proteins contribute to complex editosomes in flowering plants^5–7^. Early land plant lineages, however, seem to lack such additional components. All of the few C-to-U RNA editing sites in the moss *Physcomitrella patens* have been assigned to specific editing factors addressing one or maximally two edits, respectively^8–10^. All these editing factors are PPR proteins with a terminal DYW domain, which had been proposed to be the cytidine deaminase needed for the biochemical conversion of cytidine into uridine^11^. Further circumstantial evidence supported that concept^12–16^, but other studies questioned the idea of the DYW domain as a cytidine deaminase^17,18^.

Studying plant RNA editing in vivo is time-consuming and labor-intensive and hindered by occasionally strong mutant phenotypes, lack of a transformation protocol for plant mitochondria, and the background of interacting proteins in the native system.

Here we demonstrate that single DYW-type plant editing factors can faithfully edit their corresponding targets in *Escherichia coli*. Single amino acid mutations in the DYW domain clearly demonstrate its role as the cytidine deaminase acting on polyribonucleotides. Mutations in the cognate RNA targets and within the PLS-type PPR arrays essentially confirm the current PPR-RNA recognition code, but also reveal that some conceptually improved PPR-RNA matches may decrease rather than improve RNA editing and that at least some L-type PPRs evidently play an important role in the process. We demonstrate that changing key amino acid positions in a PPR will allow for a corresponding nucleotide change in the RNA, resulting in a designed target switch. The identification of numerous off-targets in the *E. coli* background transcriptome will accelerate our understanding of RNA target recognition by PLS-type PPRs.

## Results

### *Escherichia coli* expression system

**Fig. 1:**
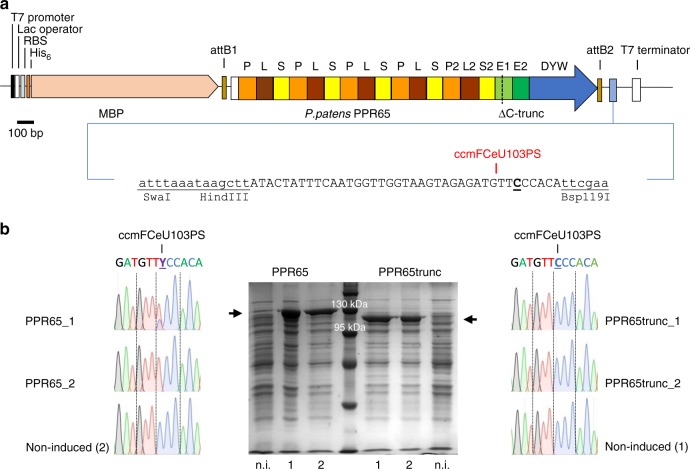
Strategy for establishing a plant C-to-U RNA editing setup in *Escherichia coli*, shown for *P. patens* pentatricopeptide repeat (PPR) protein PPR65 and its target editing site ccmFCeU103PS. **a** The PPR65 coding sequence is inserted into the pETG_41K vector system resulting in the fusion to a His_6_-tagged maltose binding protein (MBP, RBS: ribosome binding site). The editing target sequence is cloned downstream. Expression is driven by a T7 promoter inducible by isopropyl β-d-1-thiogalactopyranoside (IPTG). Editing site is labeled with target gene name (ccmFC encoding subunit FC of the cytochrome c maturation machinery) followed by eU, transcript position, and resulting amino acid change. **b** Editing of ccmFC103PS by PPR65 relies on its C-terminal domain in *E. coli*. Shown are protein expression and sequencing electropherograms revealing editing frequencies for two independent *E. coli* cultures with PPR65 and C terminally truncated PPR65, respectively, and non-induced samples as negative controls. Bacterial lysates on a denaturing sodium dodecyl sulfate-polyacrylamide gel electrophoresis (SDS-PAGE) gel correspond to ca. 4.5 × 10^7^ cells of a 20-h culture after IPTG induction (n.i. = non-induced, PPR65trunc = C terminally truncated)

The bacterial system allows for straightforward identification of the key determinants for efficient C-to-U editing both on the side of the editing protein and on the RNA target side. PPR65 has been identified as the editing factor addressing editing event ccmFCeU103PS in *P. patens* mitochondria (for nomenclature see Fig. 1) and binding to its target was previously demonstrated in vitro by RNA electromobility shift assays after successful expression in *E. coli*^8^. We adapted an expression system (see Methods), allowing insertion of target sequences behind the editing factor coding sequences on the same transcript (Fig. 1a). Indeed, isopropyl β-d-1-thiogalactopyranoside (IPTG)-induced *E. coli* cultures edited the target cytidine very efficiently from 70 up to 100% (Fig. 1b). Deleting the C terminus of PPR65, essentially reducing it to its array of 15 PLS-type PPRs, abolished C-to-U conversion completely (Fig. 1b).

### Specific nucleotides are required for RNA targeting

**Fig. 2:**
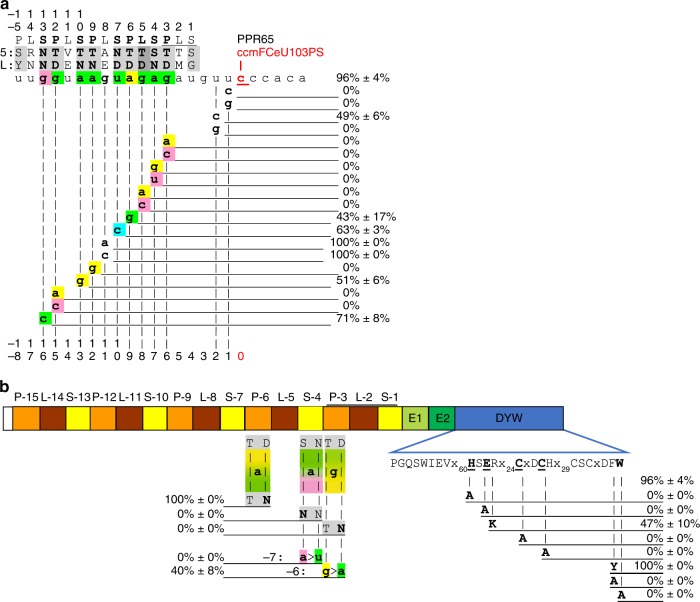
DYW-type pentatricopeptide repeat (PPR) protein PPR65 and its corresponding RNA editing site ccmFCeU103PS tested for target nucleotide (**a**) and amino acid mutations (**b**) in the new *E. coli* setup. **a** Labels of PLS-type PPRs use backward numbering (top numbers) with S-1 juxtaposed to nucleotide −4 (bottom numbers) upstream of the editing site (red) and indicate the respective amino acid identities in PPR positions 5 and L (last). The terminal P2L2S2 PPR triplet is underlined. Target nucleotide color shading is according to the PPR-RNA code for P- and S-type PPRs (gray, T/S + N: A, T/S + D: G, N + N: C/U, N + S: C > U, N + D: U > C) with green for perfect matches, blue for pyrimidine transitions, yellow for purine transitions, and pink for mismatches. Exceptionally, L-type PPR L-5TD (dark gray) would also match the G in position −8 of the native target. **b** Amino acid identities 5 or L of selected PPRs and conserved residues of the assumed Zn^2+^-binding cytidine deaminase signature and the carboxy-terminal DFW motif in the DYW domain (shown in blue) were changed as indicated. Introduced PPR mutations (left part, bold) were attempted to be compensated with corresponding mutations A-to-U and G-to-A in positions −7 and −6 in the target, respectively. RNA editing efficiencies are given as the mean ± s.d. of at least three biological replicates (independent primary *E. coli* clones) when at least some RNA editing activity was initially detected. The absence of RNA editing was confirmed with at least an additional clone. Primary data are listed in Supplementary Data 1

We created a series of RNA target mutants to check for the relevance of nucleotide identities juxtaposed with the PPR array according to the PPR-RNA recognition rules^1^ (Fig. 2a). In PPR65, six out of eight PPRs perfectly match the nucleotide recognition code for P- and S-type repeats (Fig. 2). Moreover, PPR65 exceptionally features an L-type PPR (L-5TD), which could perfectly match the expected guanosine in the target, although the functional contribution of L-type PPRs is currently not understood. All exchanges of matching purines by transversions or transitions, including the G juxtaposed with PPR L-5TD, abolish RNA editing completely except for an A-to-G change in position −13 that is moderately tolerated with reduced editing of ca. 51% (Fig. 2a). In contrast, exchange of the G in position −11 opposite of PPR L-8AE does not affect RNA editing. Converting the matching uridine in position −10 opposite of PPR S-7ND to cytidine reduces editing efficiency to ca. 63% (Fig. 2a). These data clearly underline the generally larger importance of PPRs matching purines over those matching pyrimidines. Notably, the conceptually improved matches opposite of PPR P-6TD (A-to-G) and of PPR S-13NN (G-to-C) also led to detectable reduction of editing efficiencies to ca. 43% and 71%, respectively. A complete loss of RNA editing was observed after U-to-G transversions in positions −1 and −2 upstream of the editing site. Converting a U to C in position −1 likewise abolished editing completely, whereas the same change in position −2 led to reduced editing of ca. 49% (Fig. 2a).

### Mutations in the PLS-type PPRs and a target switch

We selected PPRs P-6TD, S-4SN, and P-3TD in PPR65 for converting one of the critical residues for ribonucleotide recognition 5 or L (last) in each PPR to complement the findings for target mutations. Improving the fit of P-6TD to the A by converting it to P-6TN yields perfect editing (Fig. 2b). The native mismatch in position −9 can thus be corrected by a mutation on the protein side, but is surprisingly disfavored by an A-to-G mutation on the target side (Fig. 2a), demonstrating that yet further details of RNA recognition by editing factors remain to be elucidated. Mutating S-4SN matching an A in position −7 to S-4NN or changing P-3TD matching a G in position −6 to P-3TN both abolished editing completely. PPR65 mutant versions were combined with targets holding the appropriate nucleotides (T and A) at position −7 and −6, respectively. The target switch succeeded for the purine G-to-A transition resulting in the recovery of editing to ca. 40%, but not for the A-to-U transversion (Fig. 2b).

### DYW domain mutations confirm its cytidine deaminase function

We also exchanged key residues of the DYW domain assumed relevant for its presumed function as a cytidine deaminase, notably including those considered important for Zn^2+^ ion coordination (Fig. 2b, HxERx_24_CxxC). A single amino acid conversion into alanine at any of the important residues led to a complete loss of RNA editing. Position 4 of this signature is alternatively lysine (K) or arginine (R) in other editing factors. Converting the native arginine of PPR65 into lysine, however, reduced editing efficiency to ca. 47%. Similarly, the tyrosine (Y) of the name-giving tripeptide motif at the end of the DYW domain is occasionally replaced by phenylalanine (F), like in PPR65. The substitution of the one aromatic amino acid for the other did not result in a detectable reduction of editing efficiency (Fig. 2b). Converting the phenylalanine into alanine, however, abolishes editing activity completely, just like the mutation of the terminal tryptophan (W).

### PPR56 RNA targeting in *E. coli* reflects activity in planta

**Fig. 3:**
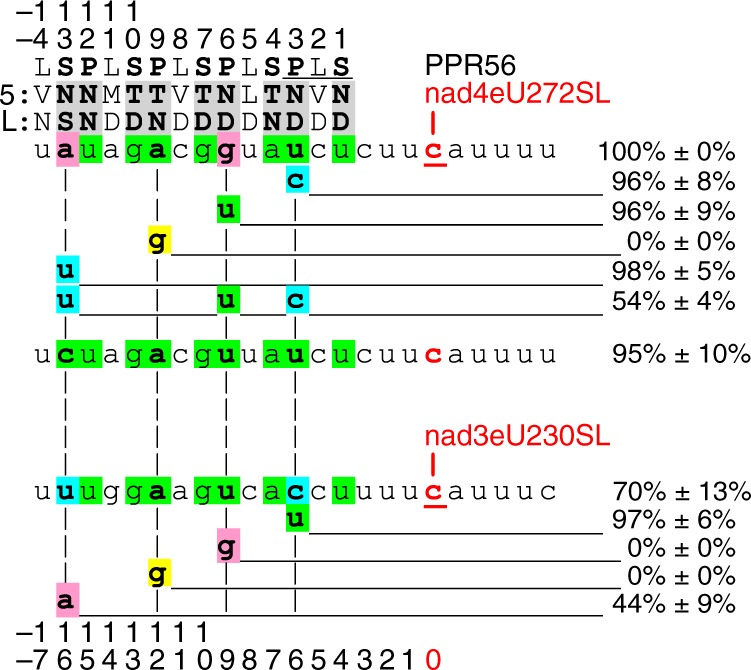
Dual-target editing factor pentatricopeptide repeat protein PPR56 expressed in the *E. coli* system with efficient editing of nad4eU272SL and partial editing of nad3eU230SL site. Designation of PPRs, numbering of positions, shading, and the scoring of editing efficiencies is as in Fig. 2. Single point mutations have been introduced into each of the two targets to match the respective other sequence. For the nad4eU272SL target, a combination of all three exchanges and a variant to fully match the PPR-RNA code concept have also been tested (middle). Additionally, the perfect match of an A in position −12 opposite of P-9TN was changed into G in both targets

We next focused on PPR56 as a second *Physcomitrella* editing factor for further studies because it serves two RNA editing targets, nad4eU272SL with 100% editing efficiency and nad3eU230SL with variably observed editing of 70–100% in planta^19–21^. The two targets were cloned alternatively behind the PPR56 coding sequence like that shown for PPR65 (Fig. 1). PPR56 in the *E. coli* setup fully reflects the native situation in the moss with partial editing detected for the nad3eU230SL target, and highly efficient editing of the nad4eU272SL target. The two alternative targets provide the option for individual mutations to be introduced for better matches to the respective other target in positions −6, −9, and −16 in the *E. coli* setup. Neither the individual changes nor the modification of the *nad4* target sequence with two point mutations for a complete match resulted in clear reductions of RNA editing in the *nad4* target after 20 h. Only introducing the mutations in positions −6, −9, and −16 jointly towards matching the *nad3* target led to reduction of the editing efficiency to ca. 54% after 20 h, indicating a cooperative effect (Fig. 3). The corresponding changes in the *nad3* target led to stronger changes and consistently follow the PPR-RNA recognition rules. The U-to-G mutation in position −9 abolishes editing, in contrast to the native *nad4* target, and the U-to-A mutation in −16 reduces editing to ca. 44%, whereas editing increases to ca. 97% with the C-to-U mutation in position −6 for improved matching to P-3ND. Purine transition A-to-G in position −12 matching P-9TN in both targets led to complete loss of editing like that observed for PPR65.

### Increase in RNA editing efficiency over time

**Fig. 4:**
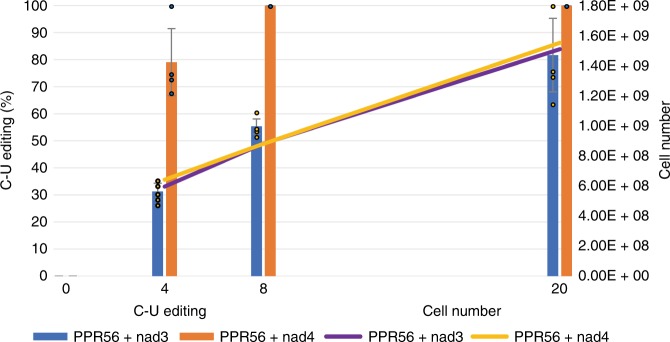
RNA editing by pentatricopeptide repeat protein PPR56 at its *nad3* and its *nad4* target increases over time. RNA editing levels and *E. coli* cell numbers were determined for dual-target editing factor PPR56 at 4, 8, and 20 h after induction of expression with 0.4 mM isopropyl β-d-1-thiogalactopyranoside (IPTG). Cell numbers were calculated based on the culture density mean at OD_600_ (s.d. 0.01–0.05) of the biological and technical replicates per time point. Three experiments with independent primary clones (biological replicates) plus 1–2 technical replicates per biological replicate were included. Dots represent individual editing data used for calculating means ± s.d. Primary data are listed in Supplementary Data 2

Given the efficient editing of the *nad4* targets in *E. coli*, we also tested shorter time periods after induction, which may allow for better differentiation (Fig. 4, Supplementary Data 1 and 2). We noticed that RNA editing efficiencies increase over time. The differences in editing of the original *nad3* and *nad4* targets were even more obvious after 4 and 8 h than after 20 h of incubation upon induction (Fig. 4, Supplementary Data 2). The strong impact of the nucleotide at position −6 (opposite of P-3ND) to editing efficiency becomes apparent after 8 h with the U-to-C change in the *nad4* target leading to a reduction of editing from 100 to 69%. Vice versa, the corresponding exchange of C to U at position −6 in the *nad3* target improves editing to 91% already after 8 h (Supplementary Data 1).

### Off-targeting in the *E. coli* system

**Fig. 5:**
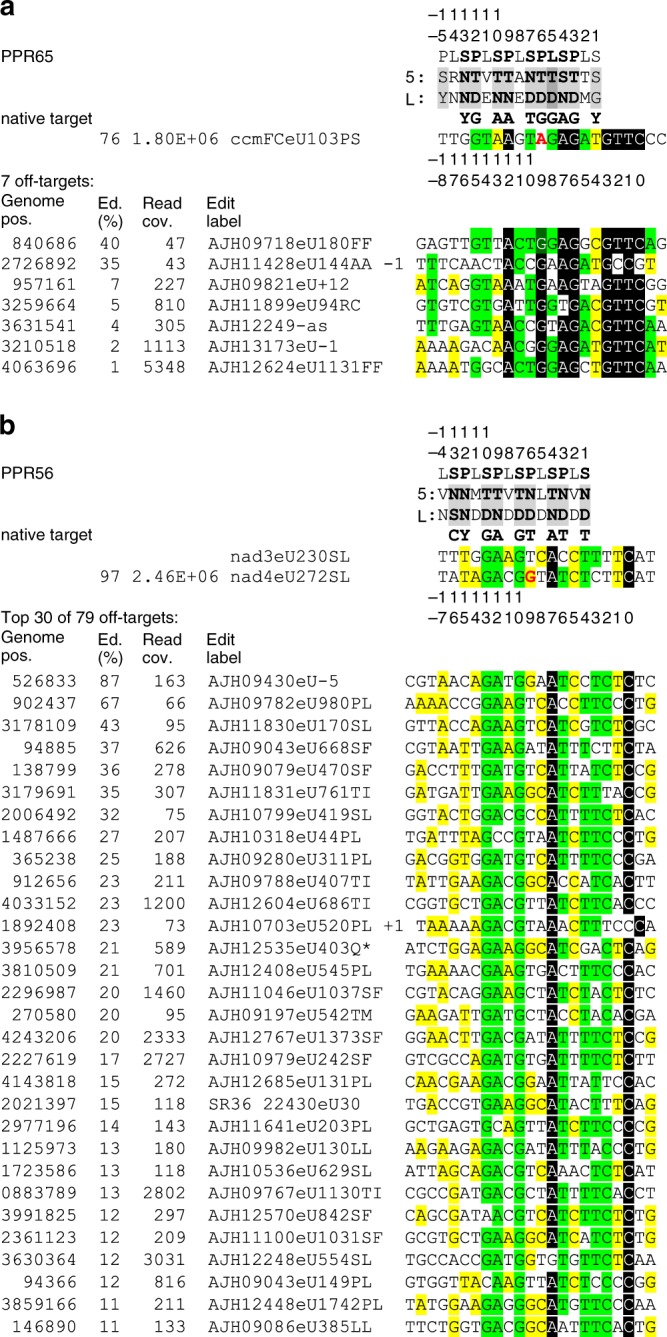
Native and off-targets of C-to-U RNA editing by pentatricopeptide repeat protein PPR65 **a** or PPR56 **b**. Off-targets are sorted with decreasing editing efficiencies and labeled using the suggested editing nomenclature (Edit label), preceded by the *E. coli* BL21 genome position (accession CP010816.1, genome pos.), average percentage of editing (Ed%), and total RNA read coverage (Read cov.) detected in two RNA-seq replicates (no positions are given for the native targets). One case of editing in antisense to an annotated gene is indicated by “as” and two cases of improved fit by one-nucleotide shifts are indicated by +1 and −1, respectively. Only the top 30 hits are shown for PPR56, the full list of off-targets is given in Supplementary Fig. 1. Shading in black, light green, and yellow indicate nucleotide conservation in 90%, 60%, or 30% of sequences. Mismatching nucleotides in native targets are highlighted in red

Evidently, the *E. coli* background transcriptome may offer secondary off-targets of C-to-U editing for the introduced plant RNA editing factors. To investigate this possibility, we performed RNA-sequencing (RNA-seq) transcriptome analyses after expression of the two editing factors. This revealed only seven off-targets for PPR65, most with editing efficiencies below 10%, but 79 sites of C-to-U editing for editing factor PPR56, with one site in a 5′-untranslated region even edited in 87% of the transcripts (Fig. 5). Comparing sequences upstream of the off-target cytidines fit expectations well with conserved nucleotide identities opposite of the relevant PPRs for the natural targets of PPR65 and PPR56, respectively. No conservation is discernible for the more upstream positions juxtaposed with further N-terminal PPRs corroborating their inferior relevance. Whereas positions −1 to −3 are identical in six of seven off-targets of PPR65, these positions are much less conserved among the PPR56 off-targets (Fig. 5). This could explain the much higher number of off-targets detected for PPR56, already targeting two editing sites in its native environment. Editing efficiencies among the off-targets overall decrease with deviations from the majority consensus. Only minor preferences occur in positions juxtaposed with L-type repeats, but in the larger PPR56 data set (Fig. 5, Supplementary Fig. 1) the majority of nucleotide always matches at least one of the native targets best. Notably, all seven off-targets of PPR65 show a G in position −9 matching the expectation for PPR P-6TD instead of the alternative A present in the native target. Likewise, it is interesting to note that G dominates in position −9 of the PPR56 off-targets, conceptually mismatched with PPR P-6ND but shared with the native, efficiently edited *nad4* target, while its less efficiently edited *nad3* counterpart features a uridine, fitting the PPR code in that position.

### Methodological issues in the *E. coli* setup

Transcript abundance, RNA secondary structures, and protein abundance are evidently among the many factors other than the linear matches of PPRs to their RNA targets that could contribute to RNA editing efficiencies. We routinely checked protein expression (Supplementary Fig. 2) and used the RNAfold WebServer^22^ to predict possible secondary structures in our targets, which did not reveal any (stable) RNA structures to be correlated with reduced or increased editing efficiencies (Supplementary Data 3). The identification of off-targets in the *E. coli* transcriptome allowed us to test whether placing a target directly behind the coding sequence of a cognate editing factor would in some, yet to be explained, way promote editing efficiency. Accordingly, we cloned AJH09430eU-5, the most efficiently edited off-target of PPR56, edited to 87% (Fig. 5), into our vector system. Rather than further increasing, RNA editing efficiencies dropped to 37% for the cloned off-target (Supplementary Data 1), confirming that the one-transcript system does not per se favor editing by factors encoded upstream on the same transcript. Individual RNA editing efficiencies may certainly be affected by numerous factors contributing to specific transcript turnover including its mode of transcription, its folding and unfolding, its processing and degradation, and the presence of RNA helicases or other RNA-binding proteins in a given genetic environment.

## Discussion

The *E. coli* RNA editing setup ultimately demonstrates that single DYW-type PPR proteins can be enough for efficient C-to-U RNA editing without any additional plant organelle-specific factors. The complete loss of RNA editing in DYW domain mutants with single amino acid changes in six independent positions (Fig. 2b) strongly supports that the DYW domain alone is the cytidine deaminase operating on RNA. It is very likely that the increasingly complex editosomes of flowering plants mainly serve to recruit functional DYW domains by (multiple) protein–protein interactions in trans when it was lost from an editing factor that became reduced to target recognition^13,23–25^. The *E. coli* setup scores ultimate editing efficiencies rather than RNA-binding efficiencies alone and seems to be more sensitive to nucleotide changes than in vitro RNA-binding studies^8,26–28^. While our study largely confirms the current concept of PPR-RNA recognition for P- and S-type PPRs, we additionally find that L-type repeats can also contribute to target recognition, at least when featuring typical residues in the crucial PPR positions 5 and L. On the other hand, we observe that RNA editing efficiency may be reduced upon conceptually improving target matches, offering a rationale for the mismatches observed for many natural editing factors and possibly indicating that RNA binding may not be too strong for efficient editing.

Single-target editing factor PPR65 seems more sensitive both to mutations in the protein and in its target sequence compared to dual-target editing factor PPR56 (Figs. 2 and 3), which is also characterized by many more off-targets in *E. coli* (Fig. 5, Supplementary Fig. 1). Similarly, dual-target editing factor CLB19 in *Arabidopsis* *thaliana* previously revealed surprising tolerance against individual mutations in planta^27^. The numerous off-targets detected upon expression of the plant RNA editing factors provide a large set of welcome extra information on cytidine target recognition. The identified off-target sequences match the proposed PPR-RNA-binding code^1^ and reveal lesser contribution of N-terminal PPR repeats to recognition^26^ (Fig. 5, Supplementary Fig. 1). The conservation of the editing site environment of the off-targets, particulary in the case of PPR65 (Fig. 5), strongly suggest nucleotide preferences also for positions directly upstream of the editing site^26^. These data will be particularly helpful to elucidate the selectivity of the E1, E2, and the DYW domains for target selection in the immediate editing site environment and ideally also for understanding a likely cooperativity between individual matches, which evidently do not simply sum up as a series of individual PPR-RNA nucleotide interactions.

The new and simple bacterial system will prove valuable for further characterization of the DYW-type RNA editing machinery, most notably so also for species currently not established as genetically accessible models^29^. The outcome of such studies will also reveal whether pyrimidine RNA editing based on PLS-type PPR proteins can be efficiently engineered to direct editing to desired sites, similar to previous approaches for tailoring of P-type PPRs for RNA binding^30,31^.

## Methods

### Cloning and expression of MBP-DYW-PPR proteins and targets

Gateway Destination vector pETG_41K (EMBL, Heidelberg, Germany, http://www.EMBL.de) was modified to obtain pET41Kmod by creating a multiple cloning site (*Swa*I, *Hin*dIII, *Asc*I, *Bsp*119I) in the former *Xho*I restriction site downstream of the gateway cassette. Protein coding sequences lacking most of the N-terminal targeting sequences were amplified with Phusion High-Fidelity DNA Polymerase (Thermo Fisher Scientific, https://www.thermofisher.com) and introduced into the Gateway pDONR/Zeo Entry vector (Thermo Fisher Scientific). Plasmid clones were used as template for mutagenesis with appropriate primers in rolling-circle PCRs^32^. Protein coding regions were transferred into pET41Kmod by Gateway cloning (Invitrogen, http://www.invitrogen.com) to create MBP fusion proteins (see Fig. 1).

RNA editing target sequences (including at least 33 bp upstream and 5 bp downstream of the editing site) flanked by appropriate restriction sites were generated by primer annealing. Twenty microliters of a mixture of 50 mM forward and reverse primer each was heated to 95 °C for 5 min and left to cool down to 40 °C. The double-stranded product was phosphorylated (T4 Polynucleotide Kinase, Thermo Fisher Scientific) and inserted into the dephosphorylated (FastAP, Thermo Fisher Scientific) pET41Kmod vector or the respective plasmid clones of editing factors. Constructs containing editing factors and downstream targets (Supplementary Data 4 and 5) were introduced into Rosetta 2 (DE3) cells (Novagen, http://www.merkmillipore.de).

For subsequent protein expression and RNA editing assays, 5 mL *E. coli* starter cultures (Luria Broth with 50 µM kanamycin and 17 µM chloramphenicol) were grown overnight. 250 µL of the pre-culture were used to inoculate 25 mL of the same media supplemented with 0.4 mM ZnSO_4_ in 100 ml Erlenmeyer flasks with baffles. Cultures were grown at 37 °C until an OD_600_ of 0.4–0.6 was reached. Cultures were cooled on ice for a minimum of 5 min before adding 0.4 mM IPTG for induction of construct expression. Cells were incubated at 16  °C and 180 rpm for 20 h before harvesting 1.5 mL samples. Shorter incubation times after induction of expression were applied test wise in selected experiments (Supplementary Data 1, Fig. 4). Samples were frozen in liquid nitrogen and stored at −80 °C until further use. Protein expression was routinely checked on denaturing sodium dodecyl sulfate-polyacrylamide gel electrophoresis gels (Fig. 1, Supplementary Figs. 2 and 3) and RNA editing was only analyzed when protein was clearly detectable.

### Detection of RNA editing in *E. coli*

To check for RNA editing in *E. coli*, complete RNA was extracted from 10^9^ cells using a kit system (Macherey and Nagel, http://www.mn-net.com) with the recommended option of a pre-incubation with lysozyme (AppliChem, https://www.applichem.com). RNA was treated with DNaseI (Thermo Fisher Scientific) and complementary DNA (cDNA) was synthesized using random hexanucleotide primers (6.4 µM per assay, Carl Roth, https://www.carlroth.com). A reverse primer upstream of the T7 terminator stem-loop sequence and a forward primer binding in the PPR protein coding region were used for reverse transcription-PCR amplification. PCR amplification assays contained 1 µL template of cDNA, 0.4 µM of each primer, 1× recommended PCR buffer, 0.8 mM dNTPs, 1U GoTaq polymerase (Promega, http://www.promega.com), and double-distilled water in a final volume of 25 µL. Amplification assays included 5 min initial denaturation at 94 °C followed by 35 cycles each with 30 s denaturation at 94 °C, 30 s annealing at 52 °C, 2.30 min synthesis at 72 °C, and a final step of synthesis for 5 min at 72 °C. Oligonucleotides are listed in Supplementary Data 6.

PCR products were gel-purified (Macherey and Nagel) and sequenced directly (Macrogen, https://dna.macrogen.com). Sequencing chromatograms were analyzed with MEGA 7 and Bioedit 7.1^33,34^ (Supplementary Data 8). RNA editing was quantified as the ratio of the resulting thymidine peak to the sum of the thymidine and cytidine peak heights at the respective editing site. Experiments were repeated with independent primary clones at least once when no editing was detected. Upon initial detection of RNA editing, at least a third independent clone was investigated given the observed variability among experiments (Supplementary Data 1). Editing values are accordingly given as the mean of at least three replicates with standard deviations as indicated in Figs. 2 and 3 and Supplementary Data 1. Designation of RNA editing sites and PPRs is according to previously suggested nomenclature proposals^35,36^ (see Figs. 1–3). RNA structure prediction was performed using RNAfold WebServer (http://rna.tbi.univie.ac.at//cgi-bin/RNAWebSuite/RNAfold.cgi) with the default settings and the temperature of 16 °C.

### RNA-seq analysis and off-target identification

To identify off-targets in the *E. coli* transcriptomes after expressing PPR56 or PPR65, respectively, total RNA was prepared as described above in two independent experiments each. Library preparation (rRNA-depleted) and Illumina sequencing (150 bp paired-end) was done by Novogene (https://en.novogene.com/).

To identify candidate sites of C-to-U editing, transcriptome reads were aligned against the *E. coli* BL21 genome (CP010816.1) in parallel with genomic DNA reads (SRX326827: SRR941832) using GSNAP^37^ v. 2017-11-15 with proposed settings^38^. RNA − DNA differences to the *E. coli* reference genome were called using JACUSA^39^. In parallel, the RNA-seq data were mapped against the pET41Kmod vector sequence with the respective PPR protein and the editing target included. For a strict identification of RNA editing sites, U vs. C or A vs. G differences in RNA vs. DNA, reads were considered only when: not filter-flagged (B, H, M, or Y) in the JACUSA output, detected in the RNA-seq data of both technical replicates, not present in RNA-seq reads of wild-type BL21 DE3 included as a control (SRX4183661: SRR7280082) or of the respective other PPR clone, and at sites showing clean DNA background (C or G > 98%) and RNA reads (T + C or G + A > 98%). The level of RNA editing was defined as the ratio of altered to total RNA reads subtracted by the same quotient for the corresponding DNA reads (nearly always zero) at each site. The original transcript mapping data are available as separate Excel sheets as Supplementary Data 7 (7-1 to 7-5).

### Reporting summary

Further information on experimental design is available in the Nature Research Reporting Summary linked to this article.

## Supplementary information

Reporting Summary

Supplementary Information

Description of Additional Supplementary Files

Supplementary Data 1

Supplementary Data 2

Supplementary Data 3

Supplementary Data 4

Supplementary Data 5

Supplementary Data 6

Supplementary Data 7

Supplementary Data 8

## Data Availability

RNA-seq data generated in this study were deposited in the NCBI Sequence Read Archive (SRA) database under the Bioproject PRJNA508474. Sanger sequence chromatograms used to score editing efficiencies are available as Supplementary Data 8.

## References

[1] Barkan A, Small I (2014). Pentatricopeptide repeat proteins in plants. Annu. Rev. Plant Biol..

[2] Oldenkott B, Yamaguchi K, Tsuji-Tsukinoki S, Knie N, Knoop V (2014). Chloroplast RNA editing going extreme: more than 3400 events of C-to-U editing in the chloroplast transcriptome of the lycophyte *Selaginella uncinata*. RNA.

[3] Cheng S (2016). Redefining the structural motifs that determine RNA binding and RNA editing by pentatricopeptide repeat proteins in land plants. Plant J..

[4] Kotera E, Tasaka M, Shikanai T (2005). A pentatricopeptide repeat protein is essential for RNA editing in chloroplasts. Nature.

[5] Sun, T., Bentolila, S. & Hanson, M. R. The unexpected diversity of plant organelle RNA editosomes. *Trends Plant Sci*. **21**, 962–973 (2016). 10.1016/j.tplants.2016.07.005 27491516

[6] Takenaka M (2012). Multiple organellar RNA editing factor (MORF) family proteins are required for RNA editing in mitochondria and plastids of plants. Proc. Natl. Acad. Sci. USA.

[7] Bentolila S (2012). RIP1, a member of an *Arabidopsis* protein family, interacts with the protein RARE1 and broadly affects RNA editing. Proc. Natl. Acad. Sci. USA.

[8] Schallenberg-Rüdinger M, Kindgren P, Zehrmann A, Small I, Knoop V (2013). A DYW-protein knockout in *Physcomitrella* affects two closely spaced mitochondrial editing sites and causes a severe developmental phenotype. Plant J..

[9] Ichinose M, Uchida M, Sugita M (2014). Identification of a pentatricopeptide repeat RNA editing factor in *Physcomitrella patens* chloroplasts. FEBS Lett..

[10] Schallenberg-Rüdinger, M. & Knoop, V. in *Genomes and Evolution of Charophytes, Bryophytes and Ferns* (ed. Rensing, S. A.) Vol. 78, 37–93 (2016).

[11] Salone V (2007). A hypothesis on the identification of the editing enzyme in plant organelles. FEBS Lett..

[12] Iyer LM, Zhang D, Rogozin IB, Aravind L (2011). Evolution of the deaminase fold and multiple origins of eukaryotic editing and mutagenic nucleic acid deaminases from bacterial toxin systems. Nucleic Acids Res..

[13] Boussardon C (2014). The cytidine deaminase signature HxE(x)nCxxC of DYW1 binds zinc and is necessary for RNA editing of ndhD-1. New Phytol..

[14] Hayes ML, Dang KN, Diaz MF, Mulligan RM (2015). A conserved glutamate residue in the C-terminal deaminase domain of pentatricopeptide repeat proteins is required for RNA editing activity. J. Biol. Chem..

[15] Rüdinger M, Volkmar U, Lenz H, Groth-Malonek M, Knoop V (2012). Nuclear DYW-type PPR gene families diversify with increasing RNA editing frequencies in liverwort and moss mitochondria. J. Mol. Evol..

[16] Wagoner JA, Sun T, Lin L, Hanson MR (2015). Cytidine deaminase motifs within the DYW domain of two pentatricopeptide repeat-containing proteins are required for site-specific chloroplast RNA editing. J. Biol. Chem..

[17] Nakamura T, Sugita M (2008). A conserved DYW domain of the pentatricopeptide repeat protein possesses a novel endoribonuclease activity. FEBS Lett..

[18] Okuda K (2009). Pentatricopeptide repeat proteins with the DYW motif have distinct molecular functions in RNA editing and RNA cleavage in *Arabidopsis* chloroplasts. Plant Cell.

[19] Ichinose M, Sugita M (2018). The DYW domains of pentatricopeptide repeat RNA editing factors contribute to discriminate target and non-target editing sites. Plant Cell Physiol..

[20] Rüdinger M, Funk HT, Rensing SA, Maier UG, Knoop V (2009). RNA editing: only eleven sites are present in the *Physcomitrella patens* mitochondrial transcriptome and a universal nomenclature proposal. Mol. Genet. Genom..

[21] Ohtani S (2010). Targeted gene disruption identifies three PPR-DYW proteins involved in RNA editing for five editing sites of the moss mitochondrial transcripts. Plant Cell Physiol..

[22] Lorenz R (2011). ViennaRNA Package 2.0. Algorithms Mol. Biol..

[23] Guillaumot D (2017). Two interacting PPR proteins are major *Arabidopsis* editing factors in plastid and mitochondria. Proc. Natl. Acad. Sci. USA.

[24] Diaz MF, Bentolila S, Hayes ML, Hanson MR, Mulligan RM (2017). A protein with an unusually short PPR domain, MEF8, affects editing at over 60 *Arabidopsis* mitochondrial C targets of RNA editing. Plant J..

[25] Andrés-Colás N (2017). Multiple PPR protein interactions are involved in the RNA editing system in *Arabidopsis* mitochondria and plastids. Proc. Natl. Acad. Sci. USA.

[26] Okuda K (2014). Quantitative analysis of motifs contributing to the interaction between PLS-subfamily members and their target RNA sequences in plastid RNA editing. Plant J..

[27] Kindgren P, Yap A, Bond CS, Small I (2015). Predictable alteration of sequence recognition by RNA editing factors from *Arabidopsis*. Plant Cell.

[28] Ramos-Vega M (2015). Functional analysis of the *Arabidopsis thaliana* chloroplast biogenesis 19 pentatricopeptide repeat editing protein. New Phytol..

[29] Schallenberg-Rüdinger M, Lenz H, Polsakiewicz M, Gott JM, Knoop V (2013). A survey of PPR proteins identifies DYW domains like those of land plant RNA editing factors in diverse eukaryotes. RNA Biol..

[30] Coquille S (2014). An artificial PPR scaffold for programmable RNA recognition. Nat. Commun..

[31] Miranda RG, McDermott JJ, Barkan A (2018). RNA-binding specificity landscapes of designer pentatricopeptide repeat proteins elucidate principles of PPR–RNA interactions. Nucleic Acids Res..

[32] Laible, M. & Boonrod, K. Homemade site directed mutagenesis of whole plasmids. *J. Vis. Exp.* **11**, 1135 (2009). 10.3791/1135 PMC2762917 19488024

[33] Kumar, S., Stecher, G. & Tamura, K. MEGA7: Molecular Evolutionary Genetics Analysis Version 7.0 for Bigger Datasets. *Mol. Biol. Evol.* **33**, 1870–1874 (2016). 10.1093/molbev/msw054 PMC8210823 27004904

[34] Hall, T. BioEdit: a user-friendly biological sequence alignment editor and analysis program for Windows 95/98/NT. *Nucleic Acids Symp. Ser.* **41**, 95–98 (1999).

[35] Lenz, H. et al. Introducing the plant RNA editing prediction and analysis computer tool PREPACT and an update on RNA editing site nomenclature. *Curr. Genet.* **56**, 189–201 (2010). 10.1007/s00294-009-0283-5 20041252

[36] Hein, A. & Knoop, V. Expected and unexpected evolution of plant RNA editing factors CLB19, CRR28 and RARE1: retention of CLB19 despite a phylogenetically deep loss of its two known editing targets in Poaceae. *BMC Evol. Biol.* **18**, 85 (2018). 10.1186/s12862-018-1203-4 PMC5992886 29879897

[37] Wu, T. D., Reeder, J., Lawrence, M., Becker, G. & Brauer, M. J. GMAP and GSNAP for Genomic Sequence Alignment: Enhancements to Speed, Accuracy, and Functionality. in *Methods in molecular biology* (Clifton, N.J.) **1418**, 283–334 (2016). 10.1007/978-1-4939-3578-9_15 27008021

[38] Picardi, E. & Pesole, G. REDItools: high-throughput RNA editing detection made easy. *Bioinformatics* **29**, 1813–1814 (2013). 10.1093/bioinformatics/btt287 23742983

[39] Piechotta, M., Wyler, E., Ohler, U., Landthaler, M. & Dieterich, C. JACUSA: site-specific identification of RNA editing events from replicate sequencing data. *BMC Bioinformatics* **18**, 7 (2017). 10.1186/s12859-016-1432-8 PMC5210316 28049429

