# Supplementary material for: Plant-type pentatricopeptide repeat proteins with a DYW domain drive C-to-U RNA editing in Escherichia coli
Source: Commun Biol. 2019 Mar 1;2:85. doi: 10.1038/s42003-019-0328-3 (PMC6397227; doi:10.1038/s42003-019-0328-3)
Supplement: Supplementary file 1 — Reporting Summary [file 42003_2019_328_MOESM1_ESM.pdf]

nature research

Corresponding author(s): Volker Knoop and Mareike Schallenberg-Rüdinger

Reporting Summary

Nature Research wishes to improve the reproducibility of the work that we publish. This form provides structure for consistency and transparency in reporting. For further information on Nature Research policies, see Authors & Referees and the Editorial Policy Checklist.

Statistical parameters

When statistical analyses are reported, confirm that the following items are present in the relevant location (e.g. figure legend, table legend, main text, or Methods section).

n/a	Confirmed	
☐	☒	The exact sample size (n) for each experimental group/condition, given as a discrete number and unit of measurement
☐	☒	An indication of whether measurements were taken from distinct samples or whether the same sample was measured repeatedly
☒	☐	The statistical test(s) used AND whether they are one- or two-sided<br>*Only common tests should be described solely by name; describe more complex techniques in the Methods section.*
☒	☐	A description of all covariates tested
☒	☐	A description of any assumptions or corrections, such as tests of normality and adjustment for multiple comparisons
☐	☒	A full description of the statistics including central tendency (e.g. means) or other basic estimates (e.g. regression coefficient) AND variation (e.g. standard deviation) or associated estimates of uncertainty (e.g. confidence intervals)
☒	☐	For null hypothesis testing, the test statistic (e.g. F, t, r) with confidence intervals, effect sizes, degrees of freedom and P value noted<br>*Give P values as exact values whenever suitable.*
☒	☐	For Bayesian analysis, information on the choice of priors and Markov chain Monte Carlo settings
☒	☐	For hierarchical and complex designs, identification of the appropriate level for tests and full reporting of outcomes
☒	☐	Estimates of effect sizes (e.g. Cohen's d, Pearson's r), indicating how they were calculated
☐	☒	Clearly defined error bars<br>*State explicitly what error bars represent (e.g. SD, SE, CI)*

Our web collection on statistics for biologists may be useful.

Software and code

Policy information about availability of computer code

Data collection	PCR products were gel-purified (Macherey and Nagel) and sequenced directly (Macrogen, https://dna.macrogen.com). Library preparation (rRNA-depleted) and Illumina sequencing (150bp paired-end) was done by Novogene (https://en.novogene.com/)
Data analysis	Sequencing chromatograms were analyzed with MEGA 7 and Bioedit 7.1. Transcriptome reads were aligned against the E. coli BL21 genome (CP010816.1) in parallel with genomic DNA reads (SRX326827: SRR941832) using GSNAP6 v. 2017-11-15 with proposed settings. RNA-DNA differences to the E. coli reference genome were called using JACUSA.

For manuscripts utilizing custom algorithms or software that are central to the research but not yet described in published literature, software must be made available to editors/reviewers upon request. We strongly encourage code deposition in a community repository (e.g. GitHub). See the Nature Research guidelines for submitting code & software for further information.

Data

Policy information about availability of data

All manuscripts must include a data availability statement. This statement should provide the following information, where applicable:

- Accession codes, unique identifiers, or web links for publicly available datasets
- A list of figures that have associated raw data
- A description of any restrictions on data availability

RNA-Seq data generated in this study were deposited in the NCBI Sequence Read Archive (SRA) database under the Bioproject PRJNA508474.
Sanger sequence chromatograms used to score editing efficiencies are available as Supplementary Data 8.

Field-specific reporting

Please select the best fit for your research. If you are not sure, read the appropriate sections before making your selection.

☒ Life sciences ☐ Behavioural & social sciences ☐ Ecological, evolutionary & environmental sciences

For a reference copy of the document with all sections, see nature.com/authors/policies/ReportingSummary-flat.pdf

Life sciences study design

All studies must disclose on these points even when the disclosure is negative.

Sample size	Experiments were repeated with independent primary clones at least once when no editing was detected. Upon initial detection of RNA editing, at least a third independent clone was investigated given the observed variability among experiments (Supplementary Data 1). Editing values are accordingly given as the mean of at least three replicates with standard deviations as indicated.<br><br>To identify off-targets in the E. coli transcriptomes after expressing PPR56 or PPR65, respectively, total RNA was prepared as described above in two independent experiments each.
Data exclusions	No data were excluded.
Replication	All attempts at replication were successful.
Randomization	Allocation was random.
Blinding	Three investigators performed experiments independently.

Reporting for specific materials, systems and methods

Materials & experimental systems

n/a	Involved in the study
☐	☒ Unique biological materials
☒	☐ Antibodies
☒	☐ Eukaryotic cell lines
☒	☐ Palaeontology
☒	☐ Animals and other organisms
☒	☐ Human research participants

Methods

n/a	Involved in the study
☒	☐ ChIP-seq
☒	☐ Flow cytometry
☒	☐ MRI-based neuroimaging

Unique biological materials

Policy information about availability of materials

Obtaining unique materials	All unique materials used are readily available from the authors upon request.
