# Supplementary material for: Plant-type pentatricopeptide repeat proteins with a DYW domain drive C-to-U RNA editing in Escherichia coli
Source: Commun Biol. 2019 Mar 1;2:85. doi: 10.1038/s42003-019-0328-3 (PMC6397227; doi:10.1038/s42003-019-0328-3)
Supplement: Supplementary file 2 — Supplementary Information [file 42003_2019_328_MOESM2_ESM.pdf]

Supplementary Information

Supplementary Figures

```
                                                      -11111
                                                      -43210987654321
PPR56                                                  LSPLSPLSPLSPLS
                                                     5:VNNMTTVTNLTNVN
                                                     L:NSNDDNDDDDNDDD
native targets                                           CY GA GT AT T
                                    nad3eU230SL        TTTGGAAGTCACCTTTTCAT
             97 2.46E+06            nad4eU272SL        TATAGACGGTATCTCTTCAT
                                                      -11111111
All 79 off-targets:                                   -765432109876543210
```

Genome pos.	Ed. [%]	Read cov.	Edit label	
73332	3	672	AJH13041eU141LL	GGAATGAAAGGTTATATTCTCAA
94366	12	816	AJH09043eU149PL	GTGGTTACAAGTTATCTCCCCGG
94885	37	626	AJH09043eU668SF	CGTAATTGAAGATATTTCTTCTA
129483	2	795	AJH09074eU984HH	GAAAACGACGCTTATGTTCACGC
138799	36	278	AJH09079eU470SF	GACCTTTGATGTCATTATCTCCG
146890	11	133	AJH09086eU385LL	TTCTGGTGACGGCAATTTCACTG
190475	9	231	AJH09124eU1019AV	GTTCGATGAAGCCATCCTCGCCC
270580	20	95	AJH09197eU542TM	GAAGATTGATGCTACCTACACGA
365238	25	188	AJH09280eU311PL	GACGGTGGATGTCATTTTCCCGA
375322	10	128	AJH13053eU100PS	TAGCCTCGATGGCATTTACCCCA
423469	3	1517	AJH09340eU653PL	CACCATCGACGTGACCTTCCCGG
477438	5	314	AJH09387eU175LL	CTACTGAGATGGCATTTCGTCTG
526833	87	163	AJH09430eU-5	CGTAACAGATGGAATCCTCTCTC
623165	8	296	AJH09525eU1161FF	ATCCCGGAAGGTAACTTCTTCGG
740583	6	192	AJH09626 as	TAGCAGACGTTTTATCTTCTCGC
883789	13	2802	AJH09767eU1130TI	CGCCGATGACGCTATTTTCACCT
896373	2	6150	AJH09777eU86AV	CGGCGGCGAAGATATTTTCGCTC
902437	67	66	AJH09782eU980PL	AAAACCGGAAGTCACCTTCCCTG
912656	23	211	AJH09788eU407TI	TATTGAAGACGGCACCATCACTT
937766	1	5236	AJH09807eU1517TI	CGAAGTTGAAGCTAAATTCACCG
958919	5	1944	AJH09823eU1046SF	CAAACAGAACGGCATGTTCTCCT
1058523	6	1399	AJH13078eU-193	TCCTCCGGTAGTCATCTCGTCAC
1125973	13	180	AJH09982eU130LL	AAGAAGAGACGATATTTACCCTG
1145454	8	2273	AJH09999eU269SL	TCATCAGGACATTATCTCCTCGC
1162861	2	2586	AJH10015eU120LL	AAAGAAGCCGATTATTATCTCAA
1272016	6	399	AJH10120eU1157TI	CGGCGATAAAGACATCTTCACCG
1387170	9	1130	AJH10234eU398SF	TACCCGTGACGCTAACTTCTCTC
1487666	27	207	AJH10318eU44PL	TGATTTAGCCGTAATCTTCCCTG
1489442	6	222	AJH10319eU949HY	TACACTGGGCGTCATCTTCCCAC
1489581	8	228	AJH10319eU810LL	CAGCTTGAAGGCAATATTCTCGC
**1582815**	**2**	**3861**	**AJH10409eU112RC**	TCTCGCGTATCGATCTTTCCCGT
**1582816**	**10**	**3851**	**AJH10409eU111SS**	GTCTCGCGTATCGATCTTTCCCG
1604568	2	1329	AJH10430eU355Q*	TCCCGTCGATGGCATCAATTCAA
1670907	6	792	AJH10490eU776AV	GGTAGAAGAAGTTATCTTCGCCC
1723586	13	118	AJH10536eU629SL	ATTAGCAGACGTCAAACTCTCAT
1859712	6	178	AJH10673eU728SL	GAAGCACGATGGCACCATCTCGG
1865979	8	238	AJH10679eU65PL	TTTACAAGAAGGCATCTTACCTT
1892408	23	73	AJH10703eU520Q* +1	-TAAAAAGACGTAAACTTTCCCA
1960793	8	601	AJH10757eU701TI	ATTACTGGAAGGTAAATTCACCG
2006492	32	75	AJH10799eU419SL	GGTACTGGACGCCATTTTCTCAC
2021397	15	118	SR36 22430eU30	TGACCGTGAAGGCATACTTTCAG
2042138	3	3743	AJH10825eU1152PP	ATGATTAGTCAGTATCTTCCCAA
2107487	4	462	AJH10880eU230SF	GTATGTCGATGAAATTTTCTCCG
2227619	17	2727	AJH10979eU242SF	GTCGCCAGATGTGATTTTCTCTT
2296987	20	1460	AJH11046eU1037SF	CGTACAGGAAGCTATCTACTCTC
2345035	5	369	AJH11086eU311TI	GGAATCAGAAGCCATCGTCACTA

2361123	12	209	AJH11100eU1031SF		GCGTGCTGAAGGCATCATCTCTG
2374733	4	11475	AJH11112eU773TI		CAATGAAGAAGCCATTTCTACCG
2849754	1	3482	AJH11527eU938TI		CCAGCATGAAGGCATTATCACCA
2872471	4	2554	AJH11546eU175HY		CAAAAGTGATGGTAACTTCCCAC
2977196	14	143	AJH11641eU203PL		GCTGAGTGCAGTTATCTTCCCCG
3178109	43	95	AJH11830eU170SL		GTTACCAGAAGTCATCGTCTCGC
3179691	35	307	AJH11831eU761TI		GATGATTGAAGGCATCTTTACCG
3277125	6	8222	AJH11927eU95SF		TGAACTGAAAGCGATCATCTCTG
3277358	4	7534	AJH11928eU416PL		CCGTGAGCAGATCATCTTCCCAG
3305587	2	1664	AJH11967eU599PL		CCGTCTGGACGGTGTTATCCCGG
3363579	6	4902	AJH12024eU1217SF	+1	-CGAACCGACGCCAACCTTCTCC
3409781	6	188	AJH12057eU126LL		TTTCTTGATGGCGATTTCCTCCA
3471791	1	7294	AJH12116eU131SF		CCACGTAGATGTAAACTACTCTG
3472102	8	1392	AJH12116eU+7		ACGAAGAAGAATAATCTTCCCTC
3605169	6	2363	AJH12227eU596SL		CATTAACCACGGTATCTTCTCGC
3630364	12	3031	AJH12248eU554SL		TGCCACCGATGGTGTGTTCTCAA
3630526	3	3177	AJH12248eU392SF		TGCGGAAGACGCCATTATCTCCG
3641764	9	340	AJH12258eU147LL		GCGGGCAAAAGCTATTTTCTCAA
3736688	4	1879	AJH12347eU1393Q*		TCGATAAGATGCTCTCTTCTCAG
3810509	21	701	AJH12408eU545PL		TGAAAACGAAGTGACTTTCCCAC
3859166	11	211	AJH12448eU1742PL		TATGGAAGAGGGCATGTTCCCAA
3887096	7	1317	AJH12476eU142RC		CGGTTAAGCTGGCATCTCACCGC
3923427	2	744	AJH12508eU560SF		GGCGCTGATGGGTGACTCCTCTG
3956578	21	589	AJH12535eU403Q*		ATCTGGAGAAGGCATCGACTCAG
3991825	12	297	AJH12570eU842SF		CAGCGATAACGTCATCTTCTCTG
4033152	23	1200	AJH12604eU686TI		CGGTGCTGACGTTATCTTCACCC
4143818	15	272	AJH12685eU131PL		CAACGAAGACGGAATTATTCCAC
4208858	3	656	AJH13206eU659PL		GCTGGAAGATGTCATTCTCCCCT
4209610	4	1161	AJH12742eU585HH		ACCCTGGAAGTGCATATCCACAA
4210006	3	1887	AJH12742eU189LL		GAAGATGGACTGCATTTTCTCGC
4243206	20	2333	AJH12767eU1373SF		GGAACTTGACGATATTTTCTCCG
4282671	2	523	AJH12803eU333SS		AAAGATGATTTGTATCTCTCCAG
4515459	4	404	AJH13027eU1583TI		GTTCTTCGAAGGTAACTTTACTG

Supplementary Figure 1. **All 79 off-targets of PPR56 (labelled as in figure 4) identified in the *E. coli* BL21 transcriptome sorted by genomic position (accession CP010816).** Shading in black, green and yellow indicates nucleotide conservation in 90%, 60% or 30% of sequences. The single case of an editing detected in antisense to an annotated gene is labeled 'as'. Six mRNAs (encoding proteins AJH09043, AJH10319, AJH10409, AJH12116 and AJH12742) are affected by two edits each. The case of AJH10409 is highlighted in bold with low editing in position 1582815 (2%, eU112RC) likely caused as a by-reaction of more efficient editing at the directly adjacent cytidine 1582816 (10%, eU111SS), better matching the target consensus.

PPR65 protein modifications

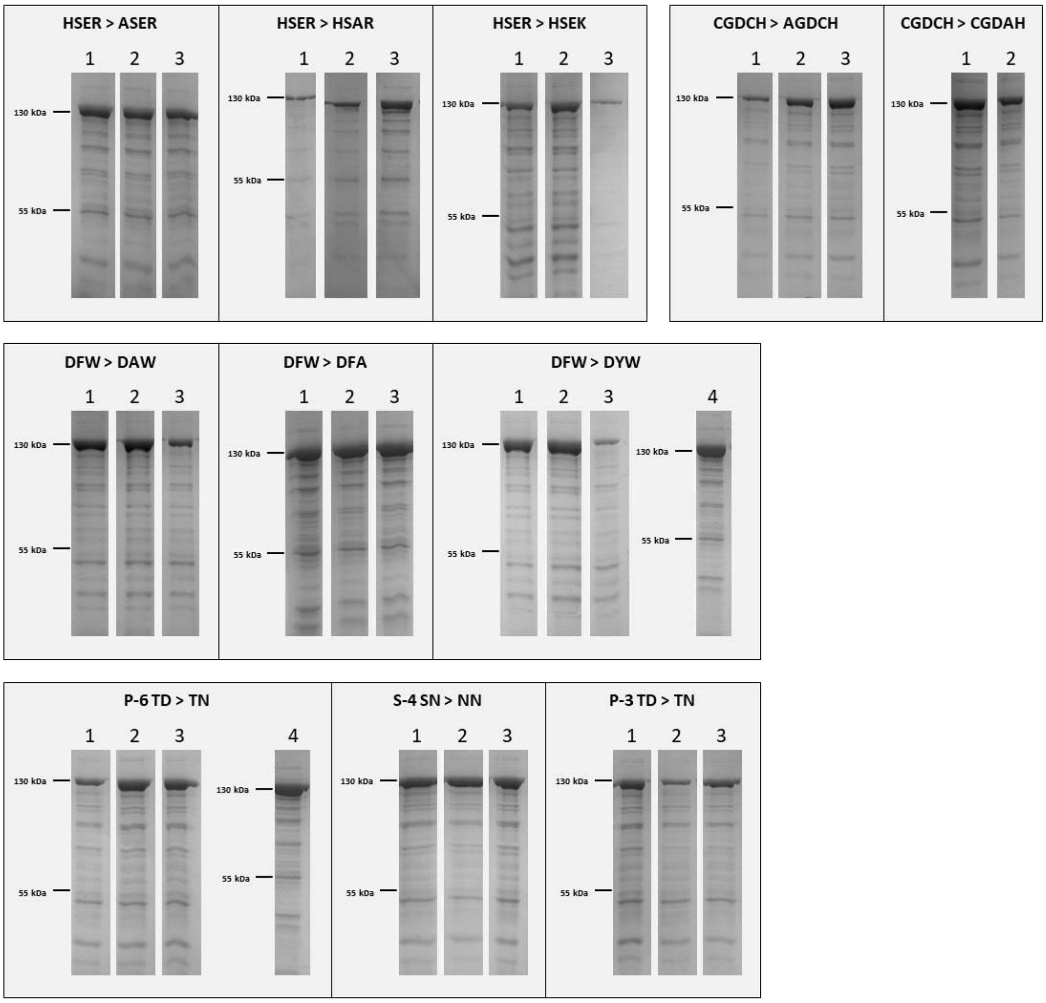

PPR56

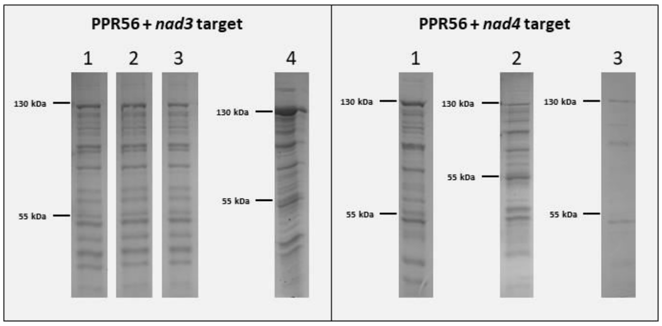

Supplementary Figure 2. Protein expression of modified versions of PPR65 and of PPR56 upstream of its two alternative targets in *E. coli.* Bacterial lysates of all biological replicates were loaded on denaturing SDS-PAGE gels (corresponding to ca. 4.5 x 10^7cells of a 20h-culture after IPTG-induction) like shown in Fig. 1B for native and truncated PPR65. Note that absence of editing was observed in many cases despite very strong expression of mutant proteins (e.g. PPR65 HSER>ASER, DFW>DFA, S-4SN>NN). *Vice versa*, 100% editing was observed even upon low amounts of detectable protein

(most notably PPR56+nad4 replicate 3 or PPR65 DFW>DYW replicate 3). Replicate 3 of the PPR65 HSER>HSEK mutation may be one exceptional example for a correlation of lower editing (34% vs. 50-55%) with lower protein expression.

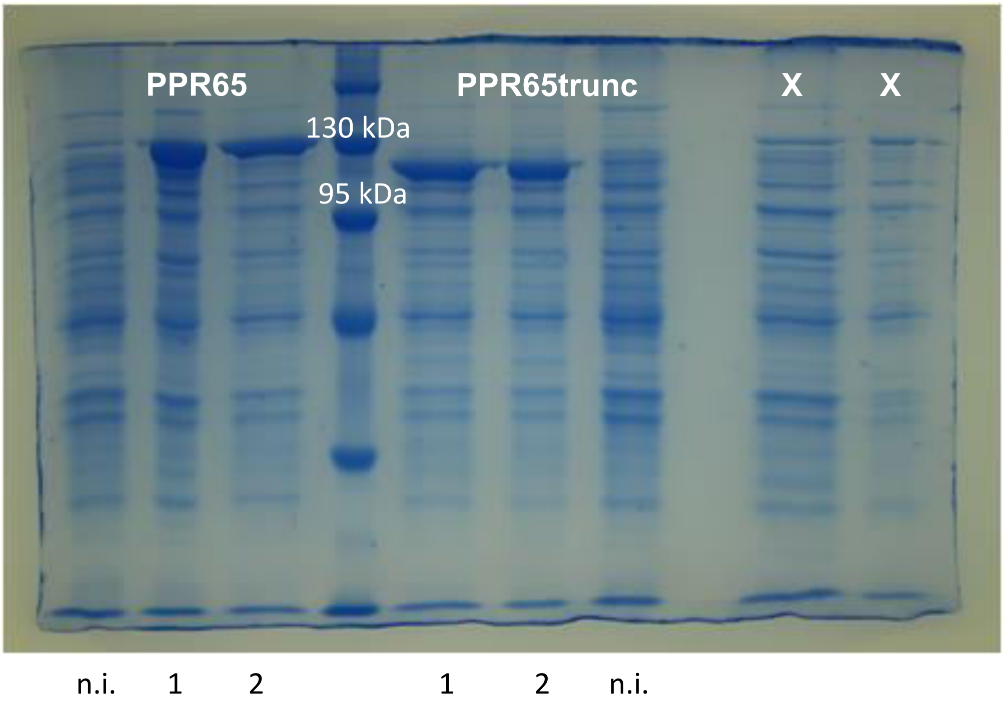

Supplementary Figure 3. Original SDS gel image presented and described in Figure 1. Shown are protein expressions for two independent *E. coli* cultures with PPR65 and C-terminally truncated PPR65, respectively and non-induced samples as negative controls. Bacterial lysates on a denaturing SDS-page gel correspond to ca. 4.5 x 10^7cells of a 20h-culture after IPTG-induction. (n.i. = non-induced, PPR65trunc = C-terminally truncated). X: protein expression of a culture not described in the publication.
