# Supplementary material for: Plant-type pentatricopeptide repeat proteins with a DYW domain drive C-to-U RNA editing in Escherichia coli
Source: Commun Biol. 2019 Mar 1;2:85. doi: 10.1038/s42003-019-0328-3 (PMC6397227; doi:10.1038/s42003-019-0328-3)
Supplement: Supplementary file 3 — Description of Additional Supplementary Files [file 42003_2019_328_MOESM3_ESM.pdf]

Description of Supplementary Data

Supplementary Data 1. Overview of PPR65 and PPR56 wild-type and mutant protein and target combinations tested for RNA editing in *Escherichia coli*. Biological replicates are defined as experiments starting from independent primary clones, technical replicates are independent experiments based on the same *E. coli* clone. RNA editing data of biological and technical replicates are listed with means ± standard deviations (s.d.). In Fig. 1 PPR65_1 and 2 represent biological replicate 3 and 4, respectively.

Supplementary Data 2. RNA editing efficiencies and culture densities (OD_{600}) of *E. coli* cultures expressing PPR56+*nad3* and PPR56+*nad*4 for 4h, 8h and 20h, respectively. Data were used to generate the bar chart in figure 4. Numbers consist of biological replicate and technical replicate. Means and standard deviations (s.d.) are calculated.

Supplementary Data 3. Potential RNA secondary structures around editing targets. RNA folding was done using the RNAfold WebServer at http://rna.tbi.univie.ac.at//cgi-bin/RNAWebSuite/RNAfold.cgi using default settings except for rescaling energy parameters to 16°C to match incubation temperature in the *E. coli* assays. Results for the Minimum Free Energy predictions (MFE in kcal mol^{-1}) are given in the common dot-bracket notation indicating unpaired and paired bases, respectively. For reference, the RNA target cloning region of the modified vector is shown on top, extending from the first nucleotide behind the protein coding region in the attB2 cloning region over the stem-loop structure (blue) of the T7 terminator (MFE -97.3 kcal mol^{-1}). The suite of restriction sites for cloning (*Swa*I-*Hin*dIII-*Asc*I-*Bsp*119I) is shown in italics. The native sequence environment in the *E. coli* genome for the top-edited off-target AJH09430eU-5 is shown at the bottom as a reference for a natural mRNA of equal length (MFE -77.9 kcal mol^{-1}). Corresponding sequences of target constructs are shown in between extending up to the last nucleotide 5' to the T7 stem-loop. Cloned targets are highlighted in bold, the assumed regions of binding the PPR arrays by underlining, the editing sites with yellow background and target sequence mutations in red font. Where RNA editing could be detected, the respective percentage is given in bold. Calculated MFEs of target constructs are consistently much closer to the natural mRNA reference of similar length (bottom) than to the vector sequence including the known RNA secondary structure of the T7 terminator (top), making the existence of comparable, stable secondary structures unlikely. Moreover, differences in potential secondary structures do not reveal obvious correlations with editing efficiency in the target mutants. For example, mutants g-8c or g-8a in the *ccmFC* target show drastic effects with no remaining editing

although not causing evident changes to a potential RNA secondary structure. Similarly, mutant a-7u with a potential secondary structure that would even be more accessible in the PPR binding region and around the editing than the PPR65 wild-type sequence showed no editing.

Supplementary Data 4. Vector pET41Kmod_PPR65_ccmFC in GeneBank file format

Supplementary Data 5. Vector pET41Kmod_PPR56_nad4 in GeneBank file format

Supplementary Data 6. List of oligonucleotides. Oligonucleotide categories are defined as follows, C: Construct design, T: Target insertion, M: Target Modification (shown is one example each of *nad3*, *nad4* and *ccmFC*), E: Editing verification, V: Vector modification, P: Protein modification; CV: construct verification, O: Off-target. Note, that oligonucleotides for native *nad3* and *nad4* targets do not reconstitute the restriction sites upon cloning into the *Swa*I and *Sgs*I sites.

Supplementary Data 7. JACUSA output file of RNA-DNA nucleotide differences identified using RNAseq reads of E*scherichia coli* clones expressing PPR proteins.

Supplementary Data 7-1: JACUSA output file of RNA-DNA nucleotide differences identified using RNAseq reads of *Escherichia coli* expressing PPR65 + *ccmFC* (biological replicate 4, technical replicate 2).

Supplementary Data 7-2: JACUSA output file of RNA-DNA nucleotide differences identified using RNAseq reads of *Escherichia coli* expressing PPR65 + *ccmFC* (biological replicate 4, technical replicate 4).

Supplementary Data 7-3: JACUSA output file of RNA-DNA nucleotide differences identified using RNAseq reads of *Escherichia coli* expressing PPR56 + *nad4* (biological replicate 1, technical replicate 3).

Supplementary Data 7-4: JACUSA output file of RNA-DNA nucleotide differences identified using RNAseq reads of *Escherichia coli* expressing PPR56 + *nad4* (biological replicate 1, technical replicate 4).

Supplementary Data 7-5: JACUSA output file of RNA-DNA nucleotide differences identified using RNAseq reads of Escherichia coli BL21 (DE3) wild type (SRX4183661: SRR7280082).

Supplementary Data 8. **Collection of Sanger sequence chromatograms used to score editing efficiencies in *E. coli* clones in the AB1 format.**
