# Supplementary material for: Plant-type pentatricopeptide repeat proteins with a DYW domain drive C-to-U RNA editing in Escherichia coli
Source: Commun Biol. 2019 Mar 1;2:85. doi: 10.1038/s42003-019-0328-3 (PMC6397227; doi:10.1038/s42003-019-0328-3)
Supplement: Supplementary file 6 — Supplementary Data 3 [file 42003_2019_328_MOESM6_ESM.docx]

>attB2 - Polylinker – T7 Terminator 97.3 kcal mol^-1^
GACCCAGCUUUCUUGUACAAAGUGGUGACUCGA*AUUUAAAUAAGCUUGGCGCGCCUUCGAA*GCACCACCACCACCACCACUGAGAUCCGGCUGCUAACAAAGCCCGAAAGGAAGCUGAGUUGGCUGCUGCCACCGCUGAGCAAUAACUAGCAU**AACCCCUUGGGGCCUCUAAACGGGUCUUGAGGGGUU**(((.((((((((((.......((((((..(((((.........((.....))...)))))..)))))).................((.((((........)))).)))))))))))).)))(((....)))...(((.((......)))))..**(((((((((((((((......)))))))))))))))**

>ccmFCeU103PS -79.2 kcal mol^-1^ **94%**
GACCCAGCUUUCUUGUACAAAGUGGUGACUCGA*AUUUAAAUAAGCUU***AUACUAUUUCAAUGGUUGGUAAGUAGAGAUGUUCCCACA***UUCGAA*UCGAGCACCACCACCACCACCACUGAGAUCCGGCUGCUAACAAAGCCCGAAAGGAAGCUGAGUUGGCUGCUGCCACCGCUGAGCAAUAACUAGCAU
(((.((((((((((.......((((((.(((((.(((......(((((((((((....)))))..))))))...(((((....))))).)))))))))))))).................((.((((........)))).)))))))))))).)))(((....)))...(((.((......)))))..

>ccmFCeU103PS u-1g -79.7 kcal mol^-1^
GACCCAGCUUUCUUGUACAAAGUGGUGACUCGA*AUUUAAAUAAGCUU***AUACUAUUUCAAUGGUUGGUAAGUAGAGAUGUGCCCACA***UUCGAA*UCGAGCACCACCACCACCACCACUGAGAUCCGGCUGCUAACAAAGCCCGAAAGGAAGCUGAGUUGGCUGCUGCCACCGCUGAGCAAUAACUAGCAU
(((.((((((((((.......((((((.(((((.(((......(((((((((((....)))))..))))))...(((((....))))).)))))))))))))).................((.((((........)))).)))))))))))).)))(((....)))...(((.((......)))))..

>ccmFCeU103PS u-1c -79.4 kcal mol^-1^
GACCCAGCUUUCUUGUACAAAGUGGUGACUCGA*AUUUAAAUAAGCUU***AUACUAUUUCAAUGGUUGGUAAGUAGAGAUGUCCCCACA***UUCGAA*UCGAGCACCACCACCACCACCACUGAGAUCCGGCUGCUAACAAAGCCCGAAAGGAAGCUGAGUUGGCUGCUGCCACCGCUGAGCAAUAACUAGCAU
(((.((((((((((.......((((((.(((((.(((......(((((((((((....)))))..))))))...(((((....))))).)))))))))))))).................((.((((........)))).)))))))))))).)))(((....)))...(((.((......)))))..

>ccmFCeU103PS u-2g -80.4 kcal mol^-1^
GACCCAGCUUUCUUGUACAAAGUGGUGACUCGA*AUUUAAAUAAGCUU***AUACUAUUUCAAUGGUUGGUAAGUAGAGAUGGUCCCACA***UUCGAA*UCGAGCACCACCACCACCACCACUGAGAUCCGGCUGCUAACAAAGCCCGAAAGGAAGCUGAGUUGGCUGCUGCCACCGCUGAGCAAUAACUAGCAU
(((.((((((((((.......((((((.(((((.(((......(((((((((((....)))))..))))))...((((......)))).)))))))))))))).................((.((((........)))).)))))))))))).)))(((....)))...(((.((......)))))..

>ccmFCeU103PS u-2c -78.7 kcal mol^-1^ **49%**
GACCCAGCUUUCUUGUACAAAGUGGUGACUCGA*AUUUAAAUAAGCUU***AUACUAUUUCAAUGGUUGGUAAGUAGAGAUGCUCCCACA***UUCGAA*UCGAGCACCACCACCACCACCACUGAGAUCCGGCUGCUAACAAAGCCCGAAAGGAAGCUGAGUUGGCUGCUGCCACCGCUGAGCAAUAACUAGCAU
(((.((((((((((.......((((((.(((((.(((......(((((((((((....)))))..))))))...((((......)))).)))))))))))))).................((.((((........)))).)))))))))))).)))(((....)))...(((.((......)))))..

>ccmFCeU103PS g-6c -78.9 kcal mol^-1^
GACCCAGCUUUCUUGUACAAAGUGGUGACUCGA*AUUUAAAUAAGCUU***AUACUAUUUCAAUGGUUGGUAAGUAGACAUGUUCCCACA***UUCGAA*UCGAGCACCACCACCACCACCACUGAGAUCCGGCUGCUAACAAAGCCCGAAAGGAAGCUGAGUUGGCUGCUGCCACCGCUGAGCAAUAACUAGCAU
(((.((((((((((.......((((((.(((((.(((......(((((((((((....)))))..)))))).((.((((....)))))))))))))))))))).................((.((((........)))).)))))))))))).)))(((....)))...(((.((......)))))..

>ccmFCeU103PS g-6a -78.9 kcal mol^-1^
GACCCAGCUUUCUUGUACAAAGUGGUGACUCGA*AUUUAAAUAAGCUU***AUACUAUUUCAAUGGUUGGUAAGUAGAAAUGUUCCCACA***UUCGAA*UCGAGCACCACCACCACCACCACUGAGAUCCGGCUGCUAACAAAGCCCGAAAGGAAGCUGAGUUGGCUGCUGCCACCGCUGAGCAAUAACUAGCAU
(((.((((((((((.......((((((.(((((.(((......(((((((((((....)))))..)))))).(((.(((....)))))))))))))))))))).................((.((((........)))).)))))))))))).)))(((....)))...(((.((......)))))..

>ccmFCeU103PS a-7u -80.1 kcal mol^-1^
GACCCAGCUUUCUUGUACAAAGUGGUGACUCGA*AUUUAAAUAAGCUU***AUACUAUUUCAAUGGUUGGUAAGUAGUGAUGUUCCCACA***UUCGAA*UCGAGCACCACCACCACCACCACUGAGAUCCGGCUGCUAACAAAGCCCGAAAGGAAGCUGAGUUGGCUGCUGCCACCGCUGAGCAAUAACUAGCAU
(((.((((((((((.......((((((.(((((.(((.(((..(((((((((((....)))))..)))))).(((.......)))))).)))))))))))))).................((.((((........)))).)))))))))))).)))(((....)))...(((.((......)))))..

>ccmFCeU103PS a-7g -83.8 kcal mol^-1^
GACCCAGCUUUCUUGUACAAAGUGGUGACUCGA*AUUUAAAUAAGCUU***AUACUAUUUCAAUGGUUGGUAAGUAGGGAUGUUCCCACA***UUCGAA*UCGAGCACCACCACCACCACCACUGAGAUCCGGCUGCUAACAAAGCCCGAAAGGAAGCUGAGUUGGCUGCUGCCACCGCUGAGCAAUAACUAGCAU
(((.((((((((((.......((((((.(((((.(((.(((..(((((((((((....)))))..)))))).((((...))))..))).)))))))))))))).................((.((((........)))).)))))))))))).)))(((....)))...(((.((......)))))..

>ccmFCeU103PS g-8c -79.2 kcal mol^-1^
GACCCAGCUUUCUUGUACAAAGUGGUGACUCGA*AUUUAAAUAAGCUU***AUACUAUUUCAAUGGUUGGUAAGUACAGAUGUUCCCACA***UUCGAA*UCGAGCACCACCACCACCACCACUGAGAUCCGGCUGCUAACAAAGCCCGAAAGGAAGCUGAGUUGGCUGCUGCCACCGCUGAGCAAUAACUAGCAU
(((.((((((((((.......((((((.(((((.(((......(((((((((((....)))))..))))))...(((((....))))).)))))))))))))).................((.((((........)))).)))))))))))).)))(((....)))...(((.((......)))))..

>ccmFCeU103PS g-8a -79.2 kcal mol^-1^
GACCCAGCUUUCUUGUACAAAGUGGUGACUCGA*AUUUAAAUAAGCUU***AUACUAUUUCAAUGGUUGGUAAGUAAAGAUGUUCCCACA***UUCGAA*UCGAGCACCACCACCACCACCACUGAGAUCCGGCUGCUAACAAAGCCCGAAAGGAAGCUGAGUUGGCUGCUGCCACCGCUGAGCAAUAACUAGCAU
(((.((((((((((.......((((((.(((((.(((......(((((((((((....)))))..))))))...(((((....))))).)))))))))))))).................((.((((........)))).)))))))))))).)))(((....)))...(((.((......)))))..

>ccmFCeU103PS a-9g -80.7 kcal mol^-1^ **48%**
GACCCAGCUUUCUUGUACAAAGUGGUGACUCGA*AUUUAAAUAAGCUU***AUACUAUUUCAAUGGUUGGUAAGUGGAGAUGUUCCCACA***UUCGAA*UCGAGCACCACCACCACCACCACUGAGAUCCGGCUGCUAACAAAGCCCGAAAGGAAGCUGAGUUGGCUGCUGCCACCGCUGAGCAAUAACUAGCAU
(((.((((((((((.......((((((.(((((.(((.(((..(((...(((((....))))).)))..((((.((...))))))))).)))))))))))))).................((.((((........)))).)))))))))))).)))(((....)))...(((.((......)))))..

>ccmFCeU103PS u-10c -81.7 kcal mol^-1^ **63%**
GACCCAGCUUUCUUGUACAAAGUGGUGACUCGA*AUUUAAAUAAGCUU***AUACUAUUUCAAUGGUUGGUAAGCAGAGAUGUUCCCACA***UUCGAA*UCGAGCACCACCACCACCACCACUGAGAUCCGGCUGCUAACAAAGCCCGAAAGGAAGCUGAGUUGGCUGCUGCCACCGCUGAGCAAUAACUAGCAU
(((.((((((((((.......((((((.(((((.(((......(((((((((((....)))))..))))))...(((((....))))).)))))))))))))).................((.((((........)))).)))))))))))).)))(((....)))...(((.((......)))))..

>ccmFCeU103PS g-11a -74.9 kcal mol^-1^ **100%**
GACCCAGCUUUCUUGUACAAAGUGGUGACUCGA*AUUUAAAUAAGCUU***AUACUAUUUCAAUGGUUGGUAAAUAGAGAUGUUCCCACA***UUCGAA*UCGAGCACCACCACCACCACCACUGAGAUCCGGCUGCUAACAAAGCCCGAAAGGAAGCUGAGUUGGCUGCUGCCACCGCUGAGCAAUAACUAGCAU
(((.((((((((((.......((((((.(((((.(((......(((...(((((....))))).))).......(((((....))))).)))))))))))))).................((.((((........)))).)))))))))))).)))(((....)))...(((.((......)))))..

>ccmFCeU103PS g-11c -76.8 kcal mol^-1^ **100%**
GACCCAGCUUUCUUGUACAAAGUGGUGACUCGA*AUUUAAAUAAGCUU***AUACUAUUUCAAUGGUUGGUAACUAGAGAUGUUCCCACA***UUCGAA*UCGAGCACCACCACCACCACCACUGAGAUCCGGCUGCUAACAAAGCCCGAAAGGAAGCUGAGUUGGCUGCUGCCACCGCUGAGCAAUAACUAGCAU
(((.((((((((((.......((((((.(((((.....................(((((((...((.(((.......)))))..)))).)))))))))))))).................((.((((........)))).)))))))))))).)))(((....)))...(((.((......)))))..

>ccmFCeU103PS a-12g -78.8 kcal mol^-1^
GACCCAGCUUUCUUGUACAAAGUGGUGACUCGA*AUUUAAAUAAGCUU***AUACUAUUUCAAUGGUUGGUAGGUAGAGAUGUUCCCACA***UUCGAA*UCGAGCACCACCACCACCACCACUGAGAUCCGGCUGCUAACAAAGCCCGAAAGGAAGCUGAGUUGGCUGCUGCCACCGCUGAGCAAUAACUAGCAU
(((.((((((((((.......((((((.(((((.(((......(((((((((((....)))))..))))))...(((((....))))).)))))))))))))).................((.((((........)))).)))))))))))).)))(((....)))...(((.((......)))))..

>ccmFCeU103PS a-13g -79.7 kcal mol^-1^ **51%**
GACCCAGCUUUCUUGUACAAAGUGGUGACUCGA*AUUUAAAUAAGCUU***AUACUAUUUCAAUGGUUGGUGAGUAGAGAUGUUCCCACA***UUCGAA*UCGAGCACCACCACCACCACCACUGAGAUCCGGCUGCUAACAAAGCCCGAAAGGAAGCUGAGUUGGCUGCUGCCACCGCUGAGCAAUAACUAGCAU
(((.((((((((((.......((((((.(((((.(((......(((((((((((....)))))..))))))...(((((....))))).)))))))))))))).................((.((((........)))).)))))))))))).)))(((....)))...(((.((......)))))..

>ccmFCeU103PS g-15a -79.0 kcal mol^-1^
GACCCAGCUUUCUUGUACAAAGUGGUGACUCGA*AUUUAAAUAAGCUU***AUACUAUUUCAAUGGUUGAUAAGUAGAGAUGUUCCCACA***UUCGAA*UCGAGCACCACCACCACCACCACUGAGAUCCGGCUGCUAACAAAGCCCGAAAGGAAGCUGAGUUGGCUGCUGCCACCGCUGAGCAAUAACUAGCAU
(((.((((((((((.......((((((.(((((.(((......(((((((((((....)))))..))))))...(((((....))))).)))))))))))))).................((.((((........)))).)))))))))))).)))(((....)))...(((.((......)))))..

>ccmFCeU103PS g-15c -77.8 kcal mol^-1^
GACCCAGCUUUCUUGUACAAAGUGGUGACUCGA*AUUUAAAUAAGCUU***AUACUAUUUCAAUGGUUGCUAAGUAGAGAUGUUCCCACA***UUCGAA*UCGAGCACCACCACCACCACCACUGAGAUCCGGCUGCUAACAAAGCCCGAAAGGAAGCUGAGUUGGCUGCUGCCACCGCUGAGCAAUAACUAGCAU
(((.((((((((((.......((((((.(((((.(((......(((((.(((((....)))))...)))))...(((((....))))).)))))))))))))).................((.((((........)))).)))))))))))).)))(((....)))...(((.((......)))))..

>ccmFCeU103PS g-16c -79.2 kcal mol^-1^ **71%**
GACCCAGCUUUCUUGUACAAAGUGGUGACUCGA*AUUUAAAUAAGCUU***AUACUAUUUCAAUGGUUCGUAAGUAGAGAUGUUCCCACA***UUCGAA*UCGAGCACCACCACCACCACCACUGAGAUCCGGCUGCUAACAAAGCCCGAAAGGAAGCUGAGUUGGCUGCUGCCACCGCUGAGCAAUAACUAGCAU
(((.((((((((((.......((((((.(((((.(((......(((((((((((....)))))..))))))...(((((....))))).)))))))))))))).................((.((((........)))).)))))))))))).)))(((....)))...(((.((......)))))..

>nad3eU230SL -71.4 kcal mol^-1^ **70%**
GACCCAGCUUUCUUGUACAAAGUGGUGACUCGAAUUU**UCUAUUUuauuuauuauauuugauuuggaagucaccuuuucauuuCC**GCGCC*UUCGAA*GCACCACCACCACCACCACUGAGAUCCGGCUGCUAACAAAGCCCGAAAGGAAGCUGAGUUGGCUGCUGCCACCGCUGAGCAAUAACUAGCAU
(((.((((((((((.......((((((..(((((.((((((..(((..((...))..)))..)))))).....................)))))..)))))).................((.((((........)))).)))))))))))).)))(((....)))...(((.((......)))))..

>nad3eU230SL c-6u -76.0 kcal mol^-1^ **97%**
GACCCAGCUUUCUUGUACAAAGUGGUGACUCGA*AUUUAAAU***UuauuuauuauauuugauuuggaagucaUcuuuucauuuC***GGCGCGCCUUCGAA*GCACCACCACCACCACCACUGAGAUCCGGCUGCUAACAAAGCCCGAAAGGAAGCUGAGUUGGCUGCUGCCACCGCUGAGCAAUAACUAGCAU
(((.((((((((((......(((((((((((.((.((((((.((.....)).)))))).)).)).))).............((.((.........)).)).........))))))....((.((((........)))).)))))))))))).)))(((....)))...(((.((......)))))..

>nad3eU230SL u-9g -78.3 kcal mol^-1^
GACCCAGCUUUCUUGUACAAAGUGGUGACUCGA*AUUUAAAU***UuauuuauuauauuugauuuggaagGcaCcuuuucauuuC***GGCGCGCCUUCGAA*GCACCACCACCACCACCACUGAGAUCCGGCUGCUAACAAAGCCCGAAAGGAAGCUGAGUUGGCUGCUGCCACCGCUGAGCAAUAACUAGCAU
(((.((((((((((......(((((((...((((((.((((.((.....)).))))))))))((((((.((..........))...))))))................)))))))....((.((((........)))).)))))))))))).)))(((....)))...(((.((......)))))..

>nad3eU230SL a-12g -78.6 kcal mol^-1^
GACCCAGCUUUCUUGUACAAAGUGGUGACUCGA*AUUUAAAU***UuauuuauuauauuugauuuggGagucaCcuuuucauuuC***GGCGCGCCUUCGAA*GCACCACCACCACCACCACUGAGAUCCGGCUGCUAACAAAGCCCGAAAGGAAGCUGAGUUGGCUGCUGCCACCGCUGAGCAAUAACUAGCAU
(((.((((((((((....((((.((((((((.((.((((((.((.....)).)))))).))..))))))))))))......((.((.........)).))...................((.((((........)))).)))))))))))).)))(((....)))...(((.((......)))))..

>nad3eU230SL u-16a -74.4 kcal mol^-1^ **44%**
GACCCAGCUUUCUUGUACAAAGUGGUGACUCGA*AUUUAAAU***UuauuuauuauauuugauAuggaagUcaCcuuuucauuuC***GGCGCGCCUUCGAA*GCACCACCACCACCACCACUGAGAUCCGGCUGCUAACAAAGCCCGAAAGGAAGCUGAGUUGGCUGCUGCCACCGCUGAGCAAUAACUAGCAU
(((.((((((((((......(((((((....((((........)))).........((.(((((((.....))))))).))((.((.........)).))........)))))))....((.((((........)))).)))))))))))).)))(((....)))...(((.((......)))))..

>nad4eU272SL -70.0 kcal mol^-1^ **100%**
GACCCAGCUUUCUUGUACAAAGUGGUGACUCGAAUUU**ucaaacaucaauuuuuauauagguauagacgguaucucuucauuuu**CGCGCC*UUCGAA*GCACCACCACCACCACCACUGAGAUCCGGCUGCUAACAAAGCCCGAAAGGAAGCUGAGUUGGCUGCUGCCACCGCUGAGCAAUAACUAGCAU
(((.((((((((((.......((((((..((((........................((((...((.(((........))).))..))))))))..)))))).................((.((((........)))).)))))))))))).)))(((....)))...(((.((......)))))..

>nad4eU272SL u-6c -75.7 kcal mol^-1^ **96%**
GACCCAGCUUUCUUGUACAAAGUGGUGACUCGA*AUUUAAAU***aucaauuuuuauauagguauagacgguaCcucuucauuuu***GGCGCGCCUUCGAA*GCACCACCACCACCACCACUGAGAUCCGGCUGCUAACAAAGCCCGAAAGGAAGCUGAGUUGGCUGCUGCCACCGCUGAGCAAUAACUAGCAU
(((.((((((((((......(((((((......((.((((........)))).))((((((.....))))))........(((.((.........)).))).......)))))))....((.((((........)))).)))))))))))).)))(((....)))...(((.((......)))))..

>nad4eU272SL g-9u -72.6 kcal mol^-1^ **96%**
GACCCAGCUUUCUUGUACAAAGUGGUGACUCGA*AUUUaaaU***aucaauuuuuauauagguauagacgUuaucucuucauuuu***GGCGCGCCUUCGAA*GCACCACCACCACCACCACUGAGAUCCGGCUGCUAACAAAGCCCGAAAGGAAGCUGAGUUGGCUGCUGCCACCGCUGAGCAAUAACUAGCAU
(((.((((((((((.....(((.((((((((..(((((.(((........))).)))))...)).)))))).))).....(((.((.........)).)))..................((.((((........)))).)))))))))))).)))(((....)))...(((.((......)))))..

>nad4eU272SL a-12g -76.4 kcal mol^-1^
GACCCAGCUUUCUUGUACAAAGUGGUGACUCGA*AUUUaaaU***aucaauuuuuauauagguauagGcgguaucucuucauuuu***GGCGCGCCUUCGAA*GCACCACCACCACCACCACUGAGAUCCGGCUGCUAACAAAGCCCGAAAGGAAGCUGAGUUGGCUGCUGCCACCGCUGAGCAAUAACUAGCAU
(((.((((((((((.......((((((..((((......(((((.((......)).)))))(((((((..............)).)))))))))..)))))).................((.((((........)))).)))))))))))).)))(((....)))...(((.((......)))))..

>nad4eU272SL a-16u -74.3 kcal mol^-1^ **98%**
GACCCAGCUUUCUUGUACAAAGUGGUGACUCGA*AUUUaaaU***aucaauuuuuauauagguUuagacgguaucucuucauuuu***GGCGCGCCUUCGAA*GCACCACCACCACCACCACUGAGAUCCGGCUGCUAACAAAGCCCGAAAGGAAGCUGAGUUGGCUGCUGCCACCGCUGAGCAAUAACUAGCAU
(((.((((((((((......(((((((....(((((((.(((........))).))))))).((.((.....))))....(((.((.........)).))).......)))))))....((.((((........)))).)))))))))))).)))(((....)))...(((.((......)))))..

>nad4eU272SL u-6c|g-9u|a-16u -77.9 kcal mol^-1^ **54%**
GACCCAGCUUUCUUGUACAAAGUGGUGACUCGA*AUUUaaaU***aucaauuuuuauauagguUuagacgUuaCcucuucauuuu***GGCGCGCCUUCGAA*GCACCACCACCACCACCACUGAGAUCCGGCUGCUAACAAAGCCCGAAAGGAAGCUGAGUUGGCUGCUGCCACCGCUGAGCAAUAACUAGCAU
(((.((((((((((.....(((.(((((((((((((((.(((........))).))))))).)).)))))).))).....(((.((.........)).)))..................((.((((........)))).)))))))))))).)))(((....)))...(((.((......)))))..

>nad4eU272SL g-9u|a-16c -72.6 kcal mol^-1^ **96%**
GACCCAGCUUUCUUGUACAAAGUGGUGACUCGA*AUUUaaaU***aucaauuuuuauauagguCuagacgUuaucucuucauuuu***GGCGCGCCUUCGAA*GCACCACCACCACCACCACUGAGAUCCGGCUGCUAACAAAGCCCGAAAGGAAGCUGAGUUGGCUGCUGCCACCGCUGAGCAAUAACUAGCAU
(((.((((((((((.....(((.((((((((..(((((.(((........))).)))))...)).)))))).))).....(((.((.........)).)))..................((.((((........)))).)))))))))))).)))(((....)))...(((.((......)))))..

>AJH09430eU-5 cloned -79.3 kcal mol^-1^ **35%**
GACCCAGCUUUCUUGUACAAAGUGGUGACUCGA*AUUUAAAU***UCCCCUUCGACCUACGUAACAGAUGGAAUCCUCUCUCUGA***GGCGCGCCUUCGAA*GCACCACCACCACCACCACUGAGAUCCGGCUGCUAACAAAGCCCGAAAGGAAGCUGAGUUGGCUGCUGCCACCGCUGAGCAAUAACUAGCAU
(((.((((((((((......(((((((....((((....))))..((((((....(((..((((.(((....))).))))..))).....))))))............)))))))....((.((((........)))).)))))))))))).)))(((....)))...(((.((......)))))..

>AJH09430eU-5 native -77.9 kcal mol^-1^ **87%**
GAAACCUGACAGCGCGCCCCGCUUCUGACAAAAUAGGCGCAUCCCCUUCGACCUACGUAACAGAUGGAAUCCUCUCUCUGAUGGCAGCAAAGAUUAUUGACGGUAAAACGAUUGCGCAGCAGGUGCGCUCUGAAGUUGCUCAAAAAGUUCAGGCGCGUAUUGCAGCCGGACUGCGGGCACCAGGACU
....((((...((.(((.(((...(((.((..(((.((((.((......)).....((..((((.(((....))).))))...))..(((......))).((......))...(((((.....))))).(((((.((.......)).))))))))).)))))))).)))...))).))..))))...

**Supplementary Data 4. Potential RNA secondary structures around editing targets.** RNA folding was done using the RNAfold WebServer at <http://rna.tbi.univie.ac.at//cgi-bin/RNAWebSuite/RNAfold.cgi> using default settings except for rescaling energy parameters to 16°C to match incubation temperature in the *E. coli* assays. Results for the Minimum Free Energy predictions (MFE in kcal/mol) are given in the common dot-bracket notation indicating unpaired and paired bases, respectively. For reference, the RNA target cloning region of the modified vector is shown on top, extending from the first nucleotide behind the protein coding region in the attB2 cloning region over the stem-loop structure (blue) of the T7 terminator (MFE -97.3 kcal/mol). The suite of restriction sites for cloning (*Swa*I-*Hin*dIII-*Asc*I-*Bsp*119I) is shown in italics. The native sequence environment in the *E. coli* genome for the top-edited off-target AJH09430eU-5 is shown at the bottom as a reference for a natural mRNA of equal length (MFE -77.9 kcal/mol). Corresponding sequences of target constructs are shown in between extending up to the last nucleotide 5’ to the T7 stem-loop. Cloned targets are highlighted in bold, the assumed regions of binding the PPR arrays by underlining, the editing sites with yellow background and target sequence mutations in red font. Where RNA editing could be detected, the respective percentage is given in bold. Calculated MFEs of target constructs are consistently much closer to the natural mRNA reference of similar length (bottom) than to the vector sequence including the known RNA secondary structure of the T7 terminator (top), making the existence of comparable, stable secondary structures unlikely. Moreover, differences in potential secondary structures do not reveal obvious correlations with editing efficiency in the target mutants. For example, mutants g-8c or g-8a in the *ccmFC* target show drastic effects with no remaining editing although not causing evident changes to a potential RNA secondary structure. Similarly, mutant a‑7u with a potential secondary structure that would even be more accessible in the PPR binding region and around the editing than the PPR65 wild-type sequence showed no editing.
