# Supplementary material for: Plant-type pentatricopeptide repeat proteins with a DYW domain drive C-to-U RNA editing in Escherichia coli
Source: Commun Biol. 2019 Mar 1;2:85. doi: 10.1038/s42003-019-0328-3 (PMC6397227; doi:10.1038/s42003-019-0328-3)
Supplement: Supplementary file 7 — Supplementary Data 4 [file 42003_2019_328_MOESM7_ESM.pdf]

```
LOCUS       Exported                8695 bp ds-DNA     circular SYN 13-JAN-2019
DEFINITION  synthetic circular DNA
ACCESSION   .
VERSION     .
KEYWORDS    .
SOURCE      synthetic DNA construct
  ORGANISM  synthetic DNA construct
REFERENCE   1  (bases 1 to 8695)
  AUTHORS   .
  TITLE     Direct Submission
  JOURNAL   Exported Jan 13, 2019 from SnapGene Viewer 4.2.11
            http://www.snapgene.com
COMMENT     Ligation of : PPR65_LR_pETG41KMCS.xdna [8647 nt] : (Bsp119I[8503] /
            HindIII[8489]) to [42 nt] : (Uncut 5'[0] / Uncut 3'[42])
FEATURES             Location/Qualifiers
     source          1..8695
                     /organism="synthetic DNA construct"
                     /mol_type="other DNA"
     rep_origin      14..469
                     /direction=RIGHT
                     /label=f1 ori
                     /note="direction=RIGHT - label=f1 ori - note=""f1
                     bacteriophage origin of replication; arrow - indicates
                     direction of (+) strand synthesis"" - 12..467"
     CDS             complement(565..1377)
                     /product="aminoglycoside phosphotransferase"
                     /label=KanR
     rep_origin      1477..2105
                     /direction=RIGHT
                     /label=ori
                     /note="high-copy-number ColE1/pMB1/pBR322/pUC origin of
                     replication"
     misc_feature    2273..2415
                     /label=bom
                     /note="basis of mobility region from pBR322"
     CDS             complement(2517..2708)
                     /product="Rop protein, which maintains plasmids at low copy
                     number"
                     /label=rop
     CDS             complement(3517..4608)
                     /codon_start=1
                     /product="lac repressor"
                     /label=lacI
                     /note="lac repressor"
                     /translation="VVNVKPVTLYDVAEYAGVSYQTVSRVVNQASHVSAKTREKVEAAM
                     AELNYIPNRVAQQLAGKQSLLIGVATSSLALHAPSQIVAAIKSRADQLGASVVVSMVER
                     SGVEACKAAVHNLLAQRVSGLIINYPLDDQDAIAVEAACTNVPALFLDVSDQTPINSII
                     FSHEDGTRLGVEHLVALGHQQIALLAGPLSSVSARLRLAGWHKYLTRNQIQPIAEREGD
                     WSAMSGFQQTMQMLNEGIVPTAMLVANDQMALGAMRAITESGLRVGADISVVGYDDTED
                     SSCYIPPLTTIKQDFRLLGQTSVDRLLQLSQGQAVKGNQLLPVSLVKRKTTLAPNTQTA
                     SPRALADSLMQLARQVSRLESGQ"
     promoter        complement(4600..4677)
                     /label=lacI promoter
                     /note="gene=""lacI"" - label=lacI promoter -
                     complement(4598..4675)"
     promoter        4986..5004
                     /label=T7 promoter
                     /note="label=T7 promoter - note=""promoter for
                     bacteriophage T7 RNA polymerase"" - 4984..5002"
     misc_binding    5005..5027
                     /label=Lac Operator
                     /bound_moiety="label=lac operator - bound_moiety=""lac
                     repressor encoded by lacI"" - note=""The lac repressor
                     binds to the lac operator to - inhibit transcription in E.
```

```
                     coli. This inhibition can be - relieved by adding lactose
                     or - isopropyl-beta-D-thiogalactopyranoside"
     RBS             5044..5066
                     /note="note=""efficient ribosome binding site from
                     bacteriophage - T7 gene 10 (Olins and Rangwala, 1989)"" -
                     5042..5064"
     CDS             5079..5096
                     /label=6xHis
     CDS             5100..6200
                     /label=MBP
     misc_feature    6219..6243
                     /label=AttB1
     CDS             6246..6266
                     /label=TEV site
     CDS             6267..8447
                     /codon_start=1
                     /label=PPR65
                     /translation="SHDQMPGNAGFRKVETLANSRDVSVLCKTGRLKEALGIMNTMILQ
                     GTRVYSDVFRGLLQECARLRSLEQGREVHAAILKSGIQPNRYLENTLLSMYAKCGSLTD
                     ARRVFDSIRDRNIVSWTAMIEAFVAGNKNLEAFKCYETMKLAGCKPDKVTFVSLLNAFT
                     NPELLQLGQKVHMEIVEAGLELEPRVGTSLVGMYAKCGDISKARVIFDRLPEKNVVTWT
                     LLIAGYAQQGQVDVALELLETMQQAEVAPNKITFASILQGCTTPAALEHGKKVHRYIIQ
                     SGYGRELWVVNSLITMYCKCGGLEEARKLFSDLPHRDVVTWTAMVTGYAQLGFHDEAIN
                     LFRRMQQQGIKPDKMTFTSVLTSCSSPAFLQEGKRIHQQLVHAGYNLDVYLQSALVSMY
                     AKCGSMDDASLVFNQMSERNVVAWTAIITGCAQHGRCREALEYFDQMKKQGIKPDKVTF
                     TSVLSACTHVGLVEEGRKHFRSMYLDYGIKPMVEHYSCFVDLLGRAGHLEEAENVILSM
                     PFIPGPSVWGALLSACRVHSDVERGERAAENVLKLDPDDDGAYVALSSIYAAAGRYEDA
                     EKVRQVMEKRDVVKEPGQSWIEVDGKVHVFHVEDKSHPESEQIYVELGKLTEQIKEMGY
                     VPDTRFVLHDVDEEQKERILFSHSERLAITYGLMKTPPGMPIRIVKNLRVCGDCHTATK
                     FISKVVGREIIARDAQRFHHFADGVCSCGDFW"
     misc_feature    8449..8473
                     /label=AttB2
     misc_feature    8494..8535
                     /label=PPR65_ccmFCeU103PS
                     /note="note=""Fragment extracted from [42 nt] : (Uncut
                     5'[0] / Uncut 3'[42])"
     CDS             8541..8558
                     /label=6xHis
     terminator      8625..8672
                     /label=T7 terminator
                     /note="transcription terminator for bacteriophage T7 RNA
                     polymerase"
ORIGIN
        1 attggcgaat gggacgcgcc ctgtagcggc gcattaagcg cggcgggtgt ggtggttacg
       61 cgcagcgtga ccgctacact tgccagcgcc ctagcgcccg ctcctttcgc tttcttccct
      121 tcctttctcg ccacgttcgc cggctttccc cgtcaagctc taaatcgggg gctcccttta
      181 gggttccgat ttagtgcttt acggcacctc gaccccaaaa aacttgatta gggtgatggt
      241 tcacgtagtg ggccatcgcc ctgatagacg gtttttcgcc ctttgacgtt ggagtccacg
      301 ttctttaata gtggactctt gttccaaact ggaacaacac tcaaccctat ctcggtctat
      361 tcttttgatt tataagggat tttgccgatt tcggcctatt ggttaaaaaa tgagctgatt
      421 taacaaaaat ttaacgcgaa ttttaacaaa atattaacgt ttacaatttc aggtggcact
      481 tttcggggaa atgtgcgcgg aacccctatt tgtttatttt tctaaataca ttcaaatatg
      541 tatccgctca tgaattaatt cttagaaaaa ctcatcgagc atcaaatgaa actgcaattt
      601 attcatatca ggattatcaa taccatattt ttgaaaaagc cgtttctgta atgaaggaga
      661 aaactcaccg aggcagttcc ataggatggc aagatcctgg tatcggtctg cgattccgac
      721 tcgtccaaca tcaatacaac ctattaattt cccctcgtca aaaataaggt tatcaagtga
      781 gaaatcacca tgagtgacga ctgaatccgg tgagaatggc aaaagtttat gcatttcttt
      841 ccagacttgt tcaacaggcc agccattacg ctcgtcatca aaatcactcg catcaaccaa
      901 accgttattc attcgtgatt gcgcctgagc gagacgaaat acgcgatcgc tgttaaaagg
      961 acaattacaa acaggaatcg aatgcaaccg gcgcaggaac actgccagcg catcaacaat
     1021 attttcacct gaatcaggat attcttctaa tacctggaat gctgttttcc cggggatcgc
     1081 agtggtgagt aaccatgcat catcaggagt acggataaaa tgcttgatgg tcggaagagg
     1141 cataaattcc gtcagccagt ttagtctgac catctcatct gtaacatcat tggcaacgct
     1201 acctttgcca tgtttcagaa acaactctgg cgcatcgggc ttcccataca atcgatagat
```

1261 tgtcgcacct gattgcccga cattatcgcg agcccattta tacccatata aatcagcatc
1321 catgttggaa tttaatcgcg gcctagagca agacgtttcc cgttgaatat ggctcataac
1381 accccttgta ttactgttta tgtaagcaga cagttttatt gttcatgacc aaaatccctt
1441 aacgtgagtt ttcgttccac tgagcgtcag accccgtaga aaagatcaaa ggatcttctt
1501 gagatccttt ttttctgcgc gtaatctgct gcttgcaaac aaaaaaacca ccgctaccag
1561 cggtggtttg tttgccggat caagagctac caactctttt tccgaaggta actggcttca
1621 gcagagcgca gataccaaat actgtccttc tagtgtagcc gtagttaggc caccacttca
1681 agaactctgt agcaccgcct acatacctcg ctctgctaat cctgttacca gtggctgctg
1741 ccagtggcga taagtcgtgt cttaccgggt tggactcaag acgatagtta ccggataagg
1801 cgcagcggtc gggctgaacg gggggttcgt gcacacagcc cagcttggag cgaacgacct
1861 acaccgaact gagataccta cagcgtgagc tatgagaaag cgccacgctt cccgaaggga
1921 gaaaggcgga caggtatccg gtaagcggca gggtcggaac aggagagcgc acgagggagc
1981 ttccaggggg aaacgcctgg tatctttata gtcctgtcgg gtttcgccac ctctgacttg
2041 agcgtcgatt tttgtgatgc tcgtcagggg ggcggagcct atggaaaaac gccagcaacg
2101 cggccttttt acggttcctg gccttttgct ggccttttgc tcacatgttc tttcctgcgt
2161 tatcccctga ttctgtggat aaccgtatta ccgcctttga gtgagctgat accgctcgcc
2221 gcagccgaac gaccgagcgc agcgagtcag tgagcgagga agcggaagag cgcctgatgc
2281 ggtattttct ccttacgcat ctgtgcggta tttcacaccg catatatggt gcactctcag
2341 tacaatctgc tctgatgccg catagttaag ccagtataca ctccgctatc gctacgtgac
2401 tgggtcatgg ctgcgccccg acacccgcca acacccgctg acgcgccctg acgggcttgt
2461 ctgctcccgg catccgctta cagacaagct gtgaccgtct ccgggagctg catgtgtcag
2521 aggttttcac cgtcatcacc gaaacgcgcg aggcagctgc ggtaaagctc atcagcgtgg
2581 tcgtgaagcg attcacagat gtctgcctgt tcatccgcgt ccagctcgtt gagtttctcc
2641 agaagcgtta atgtctggct tctgataaag cgggccatgt taagggcggt ttttttcctgt
2701 ttggtcactg atgcctccgt gtaaggggga tttctgttca tgggggtaat gataccgatg
2761 aaacgagaga ggatgctcac gatacgggtt actgatgatg aacatgcccg gttactggaa
2821 cgttgtgagg gtaaacaact ggcggtatgg atgcggcggg accagagaaa aatcactcag
2881 ggtcaatgcc agcgcttcgt taatacagat gtaggtgttc cacagggtag ccagcagcat
2941 cctgcgatgc agatccggaa cataatggtg cagggcgctg acttccgcgt ttccagactt
3001 tacgaaacac ggaaaccgaa gaccattcat gttgttgctc aggtcgcaga cgttttgcag
3061 cagcagtcgc ttcacgttcg ctcgcgtatc ggtgattcat tctgctaacc agtaaggcaa
3121 ccccgccagc ctagccgggt cctcaacgac aggagcacga tcatgcgcac ccgtggggcc
3181 gccatgccgg cgataatggc ctgcttctcg ccgaaacgtt tggtggcggg accagtgacg
3241 aaggcttgag cgagggcgtg caagattccg aataccgcaa gcgacaggcc gatcatcgtc
3301 gcgctccagc gaaagcggtc ctcgccgaaa atgacccaga gcgctgccgg cacctgtcct
3361 acgagttgca tgataaagaa gacagtcata agtgcggcga cgatagtcat gccccgcgcc
3421 caccggaagg agctgactgg gttgaaggct ctcaagggca tcggtcgaga tcccggtgcc
3481 taatgagtga gctaacttac attaattgcg ttgcgctcac tgcccgcttt ccagtcggga
3541 aacctgtcgt gccagctgca ttaatgaatc ggccaacgcg cggggagagg cggtttgcgt
3601 attgggcgcc agggtggttt ttcttttcac cagtgagacg ggcaacagct gattgccctt
3661 caccgcctgg ccctgagaga gttgcagcaa gcggtccacg ctggtttgcc ccagcaggcg
3721 aaaatcctgt ttgatggtgg ttaacggcgg gatataacat gagctgtctt cggtatcgtc
3781 gtatcccact accgagatat ccgcaccaac gcgcagcccg gactcggtaa tggcgcgcat
3841 tgcgcccagc gccatctgat cgttggcaac cagcatcgca gtgggaacga tgccctcatt
3901 cagcatttgc atggtttgtt gaaaaccgga catggcactc cagtcgcctt cccgttccgc
3961 tatcggctga atttgattgc gagtgagata tttatgccag ccagccagac gcagacgcgc
4021 cgagacagaa cttaatgggc ccgctaacag cgcgatttgc tggtgaccca atgcgaccag
4081 atgctccacg cccagtcgcg taccgtcttc atgggagaaa ataatactgt tgatgggtgt
4141 ctggtcagag acatcaagaa ataacgccgg aacattagtg caggcagctt ccacagcaat
4201 ggcatcctgg tcatccagcg gatagttaat gatcagccca ctgacgcgtt gcgcgagaag
4261 attgtgcacc gccgctttac aggcttcgac gccgcttcgt tctaccatcg acaccaccac
4321 gctggcaccc agttgatcgg cgcgagattt aatcgccgcg acaatttgcg acggcgcgtg
4381 cagggccaga ctggaggtgg caacgccaat cagcaacgac tgtttgcccg ccagttgttg
4441 tgccacgcgg ttgggaatgt aattcagctc cgccatcgcc gcttccactt tttcccgcgt
4501 tttcgcagaa acgtggctgg cctggttcac cacgcgggaa acggtctgat aagagacacc
4561 ggcatactct gcgacatcgt ataacgttac tggtttcaca ttcaccaccc tgaattgact
4621 ctcttccggg cgctatcatg ccataccgcg aaaggttttg cgccattcga tggtgtccgg
4681 gatctcgacg ctctccctta tgcgactcct gcattaggaa gcagcccagt agtaggttga
4741 ggccgttgag caccgccgcc gcaaggaatg gtgcatgcaa ggagatggcg cccaacagtc
4801 ccccggccac ggggcctgcc accataccca cgccgaaaca agcgctcatg agcccgaagt
4861 ggcgagcccg atcttcccca tcggtgatgt cggcgatata ggcgccagca accgcacctg
4921 tggcgccggt gatgccggcc acgatgcgtc cggcgtagag gatcgagatc tcgatcccgc
4981 gaaattaata cgactcacta taggggaatt gtgagcggat aacaattccc ctctagaaat
5041 aatttgtttt aactttaaga aggagatata ccatgaaaca tcaccatcac catcacccca

5101 tgaaaatcga agaaggtaaa ctggtaatct ggattaacgg cgataaaggc tataacggtc
5161 tcgctgaagt cggtaagaaa ttcgagaaag ataccggaat taaagtcacc gttgagcatc
5221 cggataaact ggaagagaaa ttcccacagg ttgcggcaac tggcgatggc cctgacatta
5281 tcttctgggc acacgaccgc tttggtggct acgctcaatc tggcctgttg gctgaaatca
5341 ccccggacaa agcgttccag gacaagctgt atccgtttac ctgggatgcc gtacgttaca
5401 acggcaagct gattgcttac ccgatcgctg ttgaagcgtt atcgctgatt tataacaaag
5461 atctgctgcc gaacccgcca aaaacctggg aagagatccc ggcgctggat aaagaactga
5521 aagcgaaagg taagagcgcg ctgatgttca acctgcaaga accgtacttc acctggccgc
5581 tgattgctgc tgacgggggt tatgcgttca agtatgaaaa cggcaagtac gacattaaag
5641 acgtgggcgt ggataacgct ggcgcgaaag cgggtctgac cttcctggtt gacctgatta
5701 aaaacaaaca catgaatgca gacaccgatt actccatcgc agaagctgcc tttaataaag
5761 gcgaaacagc gatgaccatc aacggcccgt gggcatggtc caacatcgac accagcaaag
5821 tgaattatgg tgtaacggta ctgccgacct tcaagggtca accatccaaa ccgttcgttg
5881 gcgtgctgag cgcaggtatt aacgccgcca gtccgaacaa agagctggca aaagagttcc
5941 tcgaaaacta tctgctgact gatgaaggtc tggaagcggt taataaagac aaaccgctgg
6001 gtgccgtagc gctgaagtct tacgaggaag agttggcgaa agatccacgt attgccgcca
6061 ctatggaaaa cgcccagaaa ggtgaaatca tgccgaacat cccgcagatg tccgctttct
6121 ggtatgccgt gcgtactgcg gtgatcaacg ccgccagcgg tcgtcagact gtcgatgaag
6181 ccctgaaaga cgcgcagact cccggtagcg gtaccatgac aagtttgtac aaaaaagcag
6241 gcttagagaa tctttatttt cagggctctc acgatcagat gccagggaat gcaggctttc
6301 gcaaggtcga gaccctagcg aattctcgag atgtgagtgt tctgtgtaag acagggcgtt
6361 tgaaggaggc actggggatt atgaatacga tgattctaca aggaactcgt gtctactcag
6421 acgtatttcg aggtcttctg caagagtgtg ccagattgag atcattggaa caaggtagag
6481 aagtccatgc tgcaattttg aagagtggga ttcaacctaa tagatacctt gaaaacacgt
6541 tattgagcat gtatgctaag tgtgggagtc tgacggatgc tagacgggta ttcgatagca
6601 ttcgagatcg caatatcgtg tcctggactg cgatgattga agcatttgtt gctggaaata
6661 aaaatctaga ggccttcaag tgctatgaaa cgatgaagct ggcgggatgc aagcccgaca
6721 aggtcacgtt tgtgagcctt ctcaacgcgt ttacaaaccc ggagctgtta caactaggtc
6781 agaaggtgca catggaaatt gttgaagctg gactggagtt agaacctcga gttggtacct
6841 ctcttgttgg gatgtatgcc aagtgtggag atatttccaa ggcacgagtt atttttgaca
6901 gattaccaga aaagaacgtg gtcacgtgga ctctcttaat cgctggttac gcacagcaag
6961 gtcaagtcga cgttgctctg gagcttttgg agacgatgca gcaagcagaa gtagctccca
7021 acaaaatcac gtttgctagc attcttcaag gttgcacaac tccagcggct ctagagcatg
7081 gcaagaaggt ccacagatat atcattcaat cgggatatgg tagagaactt tgggtggtca
7141 attcgctgat taccatgtat tgcaagtgtg gaggactgga agaagcccgt aaactgttta
7201 gtgacttgcc acatcgggat gtggtcactt ggacagccat ggtcacaggc tacgctcaac
7261 taggattcca tgacgaggca attaatctct ttcggcggat gcagcagcaa ggcatcaaac
7321 cggataagat gacttttaca agcgttttga caagttgttc aagccctgca tttcttcaag
7381 aagggaaaag gatccatcaa cagctggttc atgctggata caatctggat gtctatctac
7441 aaagtgccct tgttagtatg tatgcaaaat gtggaagcat ggatgacgca agtctggtgt
7501 tcaatcagat gagtgagcgg aacgttgttg cctggactgc tataataaca ggttgtgctc
7561 agcacggacg atgcagagag gcccttgaat actttgatca gatgaaaaag caaggaataa
7621 aacccgacaa ggtaaccttc acaagcgtcc tctcagcatg cacccacgta ggtctagtgg
7681 aggaaggtcg caagcacttc cgctccatgt acctagacta tggcatcaaa cccatggttg
7741 agcactacag ctgcttcgta gaccttctgg gccgagcagg ccatttggag gaagcagaga
7801 acgttatcct cagcatgcca ttcataccgg gtccctcagt ttgggggca ttgctcagcg
7861 cttgcagggt ccacagtgac gttgaacgtg gcgagcgtgc tgcagagaat gtcctcaagc
7921 tggaccctga tgatgatgga gcttacgtcg cattgtcgag tatctacgct gctgctggcc
7981 gatatgaaga cgctgagaaa gtgcggcaag tgatggagaa aagggatgtt gtcaaggagc
8041 cggggcagag ctggattgaa gtagacggca aggtgcatgt gtttcatgtg gaggataagt
8101 cccacccgga atctgagcag atttacgtcg agctcgggaa gctaactgaa cagatcaagg
8161 agatgggtta tgtaccggac accagatttg ttctgcacga cgtggatgag gaacagaagg
8221 agcggatact tttctctcac agtgagaggc tggcgatcac ttacgggctg atgaagacac
8281 cacccggaat gcctatccgt attgtgaaga atctgcgagt gtgtggggat tgtcatacgg
8341 cgacgaagtt tatctcgaag gtggttggta gagagatcat tgccagggat gcgcagcggt
8401 ttcatcattt tgccgatgga gtatgttctt gtggggattt ttggtgagac ccagctttct
8461 tgtacaaagt ggtgactcga atttaaataa gcttatacta tttcaatggt tggtaagtag
8521 agatgttccc acattcgaag caccaccacc accaccactg agatccggct gctaacaaag
8581 cccgaaagga agctgagttg gctgctgcca ccgctgagca ataactagca taaccccttg
8641 gggcctctaa acgggtcttg aggggttttt tgctgaaagg aggaactata tccgg
//
