# Supplementary material for: Plant-type pentatricopeptide repeat proteins with a DYW domain drive C-to-U RNA editing in Escherichia coli
Source: Commun Biol. 2019 Mar 1;2:85. doi: 10.1038/s42003-019-0328-3 (PMC6397227; doi:10.1038/s42003-019-0328-3)
Supplement: Supplementary file 8 — Supplementary Data 5 [file 42003_2019_328_MOESM8_ESM.pdf]

```
LOCUS       Exported                8590 bp ds-DNA     circular SYN 13-JAN-2019
DEFINITION  synthetic circular DNA
ACCESSION   .
VERSION     .
KEYWORDS    .
SOURCE      synthetic DNA construct
  ORGANISM  synthetic DNA construct
REFERENCE   1  (bases 1 to 8590)
  AUTHORS   .
  TITLE     Direct Submission
  JOURNAL   Exported Jan 13, 2019 from SnapGene Viewer 4.2.11
            http://www.snapgene.com
COMMENT     Ligation of : PPR56_LR_pET41KMCS.xdna [8540 nt] : (SgsI[8387] /
            SmiI[8373]) to PPR56_nad4_Hy.xdna [46 nt] : (Uncut 5'[0] / Uncut
            3'[46])
FEATURES             Location/Qualifiers
     source          1..8590
                     /organism="synthetic DNA construct"
                     /mol_type="other DNA"
     rep_origin      14..469
                     /direction=RIGHT
                     /label=f1 ori
                     /note="direction=RIGHT - label=f1 ori - note=""f1
                     bacteriophage origin of replication; arrow - indicates
                     direction of (+) strand synthesis"" - 12..467"
     CDS             complement(562..1377)
                     /product="aminoglycoside phosphotransferase"
                     /label=KanR
                     /note="codon_start=1 - gene=""aph(3')-Ia"" -
                     product=""aminoglycoside phosphotransferase"" - label=KanR
                     - note=""confers resistance to kanamycin in bacteria or
                     G418 - (Geneticin(R)) in eukaryotes"" -
                     translation=""MSHIQRETSCSRPRLNSNMDADLYGYKWARDNVGQSGATIYRLYG
                      - KPDAPE"
     rep_origin      1499..2087
                     /direction=RIGHT
                     /label=ori
                     /note="high-copy-number ColE1/pMB1/pBR322/pUC origin of
                     replication"
     misc_feature    2273..2415
                     /label=bom
                     /note="basis of mobility region from pBR322"
     CDS             complement(2517..2708)
                     /product="Rop protein, which maintains plasmids at low copy
                     number"
                     /label=rop
                     /note="codon_start=1 - gene=""rop"" - product=""Rop
                     protein, which maintains plasmids at low copy - number"" -
                     label=rop -
                     translation=""MTKQEKTALNMARFIRSQTLTLLEKLNELDADEQADICESLHDHA
                      - DELYRSCLARFGDDGENL"" - complement(2515..2706)"
     CDS             complement(3517..4599)
                     /product="lac repressor"
                     /label=lacI
                     /note="codon_start=1 - gene=""lacI"" - product=""lac
                     repressor"" - label=lacI - note=""The lac repressor binds
                     to the lac operator to - inhibit transcription in E. coli.
                     This inhibition can be - relieved by adding lactose or -
                     isopropyl-beta-D-thiogalactopyranosid"
     promoter        complement(4600..4677)
                     /label=lacI promoter
                     /note="gene=""lacI"" - label=lacI promoter -
                     complement(4598..4675)"
     promoter        4986..5004
```

```
                     /label=T7 promoter
                     /note="label=T7 promoter - note=""promoter for
                     bacteriophage T7 RNA polymerase"" - 4984..5002"
     primer_bind     5005..5029
                     /label=lac operator
                     /note="label=lac operator - bound_moiety=""lac repressor
                     encoded by lacI"" - note=""The lac repressor binds to the
                     lac operator to - inhibit transcription in E. coli. This
                     inhibition can be - relieved by adding lactose or -
                     isopropyl-beta-D-thiogalactopyranoside"
     RBS             5044..5066
                     /note="note=""efficient ribosome binding site from
                     bacteriophage - T7 gene 10 (Olins and Rangwala, 1989)"" -
                     5042..5064"
     CDS             5079..5096
                     /label=6xHis
                     /note="codon_start=1 - product=""6xHis affinity tag"" -
                     label=6xHis - translation=""HHHHHH"" - 5077..5094"
     CDS             5100..6200
                     /label=MBP
                     /note="codon_start=1 - gene=""malE (mutated)"" -
                     product=""maltose binding protein from E. coli"" -
                     label=MBP - note=""This version of the gene does not encode
                     a signal - sequence, so MBP will remain in the cytosol."" -
                     translation=""MKIEEGKLVIWINGDKGYNGLAEVGKKFEKDTGI"
     primer_bind     6107..6127
                     /label=M437
     misc_feature    6219..6243
                     /label=attB1
                     6226..8356
                     /note="note=""Fragment inserted from PPR56_BP_Zeo.xdna into
                     petG_41K+MCS.gb.xdna [LR] reaction"
     misc_feature    6246..6266
                     /label=TEV site
     misc_feature    6266..8339
                     /label=PPR56
                     /note="exclude the N-terminal signal peptide"
     CDS             6267..8339
                     /codon_start=1
                     /label=PPR56
                     /translation="VVLMNRLQRGLITDSFMYVEVLKRCLKQKDLMAAKQVHDCIIKSR
                     MEQNAHVMNNLLHVYIECGRLQEARCVFDALVKKSGASWNAMIAGYVEHKHAEDAMRLF
                     REMCHEGVQPNAGTYMIILKACASLSALKWGKEVHACIRHGGLESDVRVGTALLRMYGK
                     CGSINEARRIFDNLMNHDIISWTVMIGAYAQSGNGKEAYRLMLQMEQEGFKPNAITYVS
                     ILNACASEGALKWVKRVHRHALDAGLELDVRVGTALVQMYAKSGSIDDARVVFDRMKVR
                     DVVSWNVMIGAFAEHGRGHEAYDLFLQMQTEGCKPDAIMFLSILNACASAGALEWVKKI
                     HRHALDSGLEVDVRVGTALVHMYSKSGSIDDARVVFDRMKVRNVVSWNAMISGLAQHGL
                     GQDALEVFRRMTAHGVKPDRVTFVAVLSACSHAGLVDEGRSQYLAMTQVYGIEPDVSHC
                     NCMVDLLGRAGRLMEAKLFIDNMAVDPDEATWGALLGSCRTYGNVELGELVAKERLKLD
                     PKNAATYVLLSNIYAEAGKWDMVSWVRTMMRERGIRKEPGRSWIEVDNKIHDFLVADSS
                     HPECKEINESKDKVIEKIKAEGYIPDTRLVLKNKNMKDKELDICSHSEKLAIVYGLMHT
                     PPGNPIRVFKNLRVCTDCHGATKLISKVEGREIIVRDANRFHHFKDGVCSCGDYW"
     primer_bind     7239..7258
                     /label=M1002
     misc_feature    8341..8365
                     /label=attB2
     misc_feature    8377..8422
                     /label=PPR56_nad4eU272SL
                     /note="41+5"
     CDS             8436..8453
                     /label=6xHis
                     /note="codon_start=1 - product=""6xHis affinity tag"" -
                     label=6xHis - translation=""HHHHHH"" - 7958..7975"
     primer_bind     complement(8508..8530)
```

```
                     /label=M438
     terminator      8520..8567
                     /label=T7 terminator
                     /note="transcription terminator for bacteriophage T7 RNA
                     polymerase"
ORIGIN
        1 attggcgaat gggacgcgcc ctgtagcggc gcattaagcg cggcgggtgt ggtggttacg
       61 cgcagcgtga ccgctacact tgccagcgcc ctagcgcccg ctcctttcgc tttcttccct
      121 tcctttctcg ccacgttcgc cggctttccc cgtcaagctc taaatcgggg gctcccttta
      181 gggttccgat ttagtgcttt acggcacctc gaccccaaaa aacttgatta gggtgatggt
      241 tcacgtagtg ggccatcgcc ctgatagacg gtttttcgcc ctttgacgtt ggagtccacg
      301 ttctttaata gtggactctt gttccaaact ggaacaacac tcaaccctat ctcggtctat
      361 tcttttgatt tataagggat tttgccgatt tcggcctatt ggttaaaaaa tgagctgatt
      421 taacaaaaat ttaacgcgaa ttttaacaaa atattaacgt ttacaatttc aggtggcact
      481 tttcggggaa atgtgcgcgg aacccctatt tgtttatttt tctaaataca ttcaaatatg
      541 tatccgctca tgaattaatt cttagaaaaa ctcatcgagc atcaaatgaa actgcaattt
      601 attcatatca ggattatcaa taccatattt ttgaaaaagc cgtttctgta atgaaggaga
      661 aaactcaccg aggcagttcc ataggatggc aagatcctgg tatcggtctg cgattccgac
      721 tcgtccaaca tcaatacaac ctattaattt cccctcgtca aaaataaggt tatcaagtga
      781 gaaatcacca tgagtgacga ctgaatccgg tgagaatggc aaaagtttat gcatttcttt
      841 ccagacttgt tcaacaggcc agccattacg ctcgtcatca aaatcactcg catcaaccaa
      901 accgttattc attcgtgatt gcgcctgagc gagacgaaat acgcgatcgc tgttaaaagg
      961 acaattacaa acaggaatcg aatgcaaccg gcgcaggaac actgccagcg catcaacaat
     1021 attttcacct gaatcaggat attcttctaa tacctggaat gctgttttcc cggggatcgc
     1081 agtggtgagt aaccatgcat catcaggagt acggataaaa tgcttgatgg tcggaagagg
     1141 cataaattcc gtcagccagt ttagtctgac catctcatct gtaacatcat tggcaacgct
     1201 acctttgcca tgtttcagaa acaactctgg cgcatcgggc ttcccataca atcgatagat
     1261 tgtcgcacct gattgcccga cattatcgcg agcccattta tacccatata aatcagcatc
     1321 catgttggaa tttaatcgcg gcctagagca agacgtttcc cgttgaatat ggctcataac
     1381 accccttgta ttactgttta tgtaagcaga cagttttatt gttcatgacc aaaatccctt
     1441 aacgtgagtt ttcgttccac tgagcgtcag accccgtaga aaagatcaaa ggatcttctt
     1501 gagatccttt ttttctgcgc gtaatctgct gcttgcaaac aaaaaaacca ccgctaccag
     1561 cggtggtttg tttgccggat caagagctac caactctttt tccgaaggta actggcttca
     1621 gcagagcgca gataccaaat actgtccttc tagtgtagcc gtagttaggc caccacttca
     1681 agaactctgt agcaccgcct acatacctcg ctctgctaat cctgttacca gtggctgctg
     1741 ccagtggcga taagtcgtgt cttaccgggt tggactcaag acgatagtta ccggataagg
     1801 cgcagcggtc gggctgaacg gggggttcgt gcacacagcc cagcttggag cgaacgacct
     1861 acaccgaact gagataccta cagcgtgagc tatgagaaag cgccacgctt cccgaaggga
     1921 gaaaggcgga caggtatccg gtaagcggca gggtcggaac aggagagcgc acgagggagc
     1981 ttccaggggg aaacgcctgg tatctttata gtcctgtcgg gtttcgccac ctctgacttg
     2041 agcgtcgatt tttgtgatgc tcgtcagggg ggcggagcct atggaaaaac gccagcaacg
     2101 cggccttttt acggttcctg gccttttgct ggccttttgc tcacatgttc tttcctgcgt
     2161 tatcccctga ttctgtggat aaccgtatta ccgcctttga gtgagctgat accgctcgcc
     2221 gcagccgaac gaccgagcgc agcgagtcag tgagcgagga agcggaagag cgcctgatgc
     2281 ggtattttct ccttacgcat ctgtgcggta tttcacaccg catatatggt gcactctcag
     2341 tacaatctgc tctgatgccg catagttaag ccagtataca ctccgctatc gctacgtgac
     2401 tgggtcatgg ctgcgccccg acacccgcca acacccgctg acgcgccctg acgggcttgt
     2461 ctgctcccgg catccgctta cagacaagct gtgaccgtct ccgggagctg catgtgtcag
     2521 aggttttcac cgtcatcacc gaaacgcgcg aggcagctgc ggtaaagctc atcagcgtgg
     2581 tcgtgaagcg attcacagat gtctgcctgt tcatccgcgt ccagctcgtt gagtttctcc
     2641 agaagcgtta atgtctggct tctgataaag cgggccatgt taagggcggt tttttcctgt
     2701 ttggtcactg atgcctccgt gtaaggggga tttctgttca tgggggtaat gataccgatg
     2761 aaacgagaga ggatgctcac gatacgggtt actgatgatg aacatgcccg gttactggaa
     2821 cgttgtgagg gtaaacaact ggcggtatgg atgcggcggg accagagaaa aatcactcag
     2881 ggtcaatgcc agcgcttcgt taatacagat gtaggtgttc cacagggtag ccagcagcat
     2941 cctgcgatgc agatccggaa cataatggtg cagggcgctg acttccgcgt ttccagactt
     3001 tacgaaacac ggaaaccgaa gaccattcat gttgttgctc aggtcgcaga cgttttgcag
     3061 cagcagtcgc ttcacgttcg ctcgcgtatc ggtgattcat tctgctaacc agtaaggcaa
     3121 ccccgccagc ctagccgggt cctcaacgac aggagcacga tcatgcgcac ccgtggggcc
     3181 gccatgccgg cgataatggc ctgcttctcg ccgaaacgtt tggtggcggg accagtgacg
     3241 aaggcttgag cgagggcgtg caagattccg aataccgcaa gcgacaggcc gatcatcgtc
     3301 gcgctccagc gaaagcggtc ctcgccgaaa atgacccaga gcgctgccgg cacctgtcct
     3361 acgagttgca tgataaagaa gacagtcata agtgcggcga cgatagtcat gccccgcgcc
     3421 caccggaagg agctgactgg gttgaaggct ctcaagggca tcggtcgaga tcccggtgcc
```

3481 taatgagtga gctaacttac attaattgcg ttgcgctcac tgcccgcttt ccagtcggga
3541 aacctgtcgt gccagctgca ttaatgaatc ggccaacgcg cggggagagg cggtttgcgt
3601 attgggcgcc agggtggttt ttcttttcac cagtgagacg ggcaacagct gattgccctt
3661 caccgcctgg ccctgagaga gttgcagcaa gcggtccacg ctggtttgcc ccagcaggcg
3721 aaaatcctgt ttgatggtgg ttaacggcgg gatataacat gagctgtctt cggtatcgtc
3781 gtatcccact accgagatat ccgcaccaac gcgcagcccg gactcggtaa tggcgcgcat
3841 tgcgcccagc gccatctgat cgttggcaac cagcatcgca gtgggaacga tgccctcatt
3901 cagcatttgc atggtttgtt gaaaaccgga catggcactc cagtcgcctt cccgttccgc
3961 tatcggctga atttgattgc gagtgagata tttatgccag ccagccagac gcagacgcgc
4021 cgagacagaa cttaatgggc ccgctaacag cgcgatttgc tggtgaccca atgcgaccag
4081 atgctccacg cccagtcgcg taccgtcttc atgggagaaa ataatactgt tgatgggtgt
4141 ctggtcagag acatcaagaa ataacgccgg aacattagtg caggcagctt ccacagcaat
4201 ggcatcctgg tcatccagcg gatagttaat gatcagccca ctgacgcgtt gcgcgagaag
4261 attgtgcacc gccgctttac aggcttcgac gccgcttcgt tctaccatcg acaccaccac
4321 gctggcaccc agttgatcgg cgcgagattt aatcgccgcg acaatttgcg acggcgcgtg
4381 cagggccaga ctggaggtgg caacgccaat cagcaacgac tgtttgcccg ccagttgttg
4441 tgccacgcgg ttgggaatgt aattcagctc cgccatcgcc gcttccactt tttcccgcgt
4501 tttcgcagaa acgtggctgg cctggttcac cacgcgggaa acggtctgat aagagacacc
4561 ggcatactct gcgacatcgt ataacgttac tggtttcaca ttcaccaccc tgaattgact
4621 ctcttccggg cgctatcatg ccataccgcg aaaggttttg cgccattcga tggtgtccgg
4681 gatctcgacg ctctccctta tgcgactcct gcattaggaa gcagcccagt agtaggttga
4741 ggccgttgag caccgccgcc gcaaggaatg gtgcatgcaa ggagatggcg cccaacagtc
4801 ccccggccac ggggcctgcc accataccca cgccgaaaca agcgctcatg agcccgaagt
4861 ggcgagcccg atcttcccca tcggtgatgt cggcgatata ggcgccagca accgcacctg
4921 tggcgccggt gatgccggcc acgatgcgtc cggcgtagag gatcgagatc tcgatcccgc
4981 gaaattaata cgactcacta taggggaatt gtgagcggat aacaattccc ctctagaaat
5041 aattttgttt aactttaaga aggagatata ccatgaaaca tcaccatcac catcacccca
5101 tgaaaatcga agaaggtaaa ctggtaatct ggattaacgg cgataaaggc tataacggtc
5161 tcgctgaagt cggtaagaaa ttcgagaaag ataccggaat taaagtcacc gttgagcatc
5221 cggataaact ggaagagaaa ttcccacagg ttgcggcaac tggcgatggc cctgacatta
5281 tcttctgggc acacgaccgc tttggtggct acgctcaatc tggcctgttg gctgaaatca
5341 ccccggacaa agcgttccag gacaagctgt atccgtttac ctgggatgcc gtacgttaca
5401 acggcaagct gattgcttac ccgatcgctg ttgaagcgtt atcgctgatt tataacaaag
5461 atctgctgcc gaacccgcca aaaacctggg aagagatccc ggcgctggat aaagaactga
5521 aagcgaaagg taagagcgcg ctgatgttca acctgcaaga accgtacttc acctggccgc
5581 tgattgctgc tgacgggggt tatgcgttca agtatgaaaa cggcaagtac gacattaaag
5641 acgtgggcgt ggataacgct ggcgcgaaag cgggtctgac cttcctggtt gacctgatta
5701 aaaacaaaca catgaatgca gacaccgatt actccatcgc agaagctgcc tttaataaag
5761 gcgaaacagc gatgaccatc aacggcccgt gggcatggtc caacatcgac accagcaaag
5821 tgaattatgg tgtaacggta ctgccgacct tcaagggtca accatccaaa ccgttcgttg
5881 gcgtgctgag cgcaggtatt aacgccgcca gtccgaacaa agagctggca aaagagttcc
5941 tcgaaaacta tctgctgact gatgaaggtc tggaagcggt taataaagac aaaccgctgg
6001 gtgccgtagc gctgaagtct tacgaggaag agttggcgaa agatccacgt attgccgcca
6061 ctatggaaaa cgcccagaaa ggtgaaatca tgccgaacat cccgcagatg tccgctttct
6121 ggtatgccgt gcgtactgcg gtgatcaacg ccgccagcgg tcgtcagact gtcgatgaag
6181 ccctgaaaga cgcgcagact cccggtagcg gtaccatgac aagtttgtac aaaaaagcag
6241 gcttagagaa tctttatttt cagggcgtgg ttctgatgaa ccgtctgcaa cgaggcctta
6301 ttacagattc ttttatgtat gttgaagttt tgaagcggtg cttgaagcaa aaagacctta
6361 tggcagccaa gcaggttcat gattgcatca tcaaaagcag aatggaacaa aacgcacatg
6421 tgatgaacaa cctcctgcat gtgtacatcg aatgtgggag attgcaggag gcacgctgtg
6481 tgtttgatgc acttgtgaaa aagagtggtg ctagttggaa tgccatgatc gcaggttatg
6541 tggagcacaa gcatgccgag gatgcaatga gactttttag ggaaatgtgc cacgaaggtg
6601 tgcagccaaa tgcgggtact tacatgatca tcttgaaggc ttgtgccagt ttgtccgcgt
6661 tgaaatgggg caaagaagtt catgcatgca ttagacatgg tgggcttgaa tcagatgtgc
6721 gggtggggac tgcccttctt aggatgtatg gaaagtgtgg gtccatcaac gaggcaaggc
6781 gtatttttga taacctgatg aatcatgata tcatctcctg gactgtgatg attggagctt
6841 atgcacaaag tggaaatggc aaggaagcat atcgacttat gttacaaatg gaacaggaag
6901 gattcaaacc aaatgcaatc acttatgtga gcatcttgaa cgcatgtgca agtgaggggg
6961 cattgaagtg ggtgaagagg gttcacaggc atgccttgga tgcagggctt gagttggatg
7021 tgcgtgtggg cactgcactt gttcaaatgt acgcaaagag tggtagcatt gatgatgcac
7081 gagtggtgtt tgacagaatg aaggtgcgtg atgttgtatc atggaatgtg atgataggcg
7141 catttgcaga acatgggcgt gggcatgaag catatgatct gtttcttcag atgcagacag
7201 agggctgtaa gccagatgca atcatgtttt tgagcatctt gaatgcatgt gcaagtgcag
7261 gggcattgga gtgggtgaag aagattcaca ggcatgcctt ggattcaggg cttgaggttg

```
     7321 atgtgcgtgt gggcactgca cttgttcaca tgtactcaaa gagtggtagc attgatgatg
     7381 cgcgagtggt gtttgacaga atgaaggtgc gcaatgttgt atcatggaat gcgatgattt
     7441 ctggcttggc acaacatggt cttgggcagg acgctttgga ggtttttaga cgaatgactg
     7501 cacacggtgt gaagcctgat agggtcacat ttgtagcagt tctgtcagca tgcagtcatg
     7561 caggccttgt tgatgaaggt cgttcccagt atttggctat gacacaagtt tatggcattg
     7621 aacccgatgt gtctcattgt aactgcatgg tggatctcct ggggcgggca ggacgcttga
     7681 tggaagcaaa gctcttcatt gataacatgg cagttgaccc tgatgaagca acttggggag
     7741 ccctgcttgg ttcttgtaga acttatggta acgtagagtt gggcgaactt gttgcgaaag
     7801 aacgtctgaa attggatccg aaaaatgctg ctacctatgt tctcctttcc aacatctatg
     7861 ctgaagctgg aaagtgggac atggtatcat gggtgcggac tatgatgcgc gagagaggga
     7921 ttcgcaagga gccaggacgt agctggatcg aagtcgataa caagattcat gatttttttgg
     7981 tagccgactc atctcatcca gaatgcaagg agataaatga aagtaaggac aaagtgattg
     8041 aaaaaataaa ggctgaaggg tacattcctg atacccggct agtgctgaag aacaagaaca
     8101 tgaaggacaa agaacttgat atttgctctc acagtgagaa gttggcaatt gtgtatgggc
     8161 tgatgcatac accccctgga aatcctattc gtgtattcaa gaaccttcgg gtgtgcaccg
     8221 attgtcatgg agcaactaag ttaatctcaa aggtggaggg acgagagatc atagtgaggg
     8281 atgctaatcg gtttcaccat tttaaagatg gcgtctgctc atgtggtgat tactggtgag
     8341 acccagcttt cttgtacaaa gtggtgactc gaattttcaa acatcaattt ttatataggt
     8401 atagacggta tctcttcatt ttcgcgcctt cgaagcacca ccaccaccac cactgagatc
     8461 cggctgctaa caaagcccga aaggaagctg agttggctgc tgccaccgct gagcaataac
     8521 tagcataacc ccttggggcc tctaaacggg tcttgagggg tttttttgctg aaaggaggaa
     8581 ctatatccgg
//
```
